# Comparative RNA-Seq Analysis of Differentially Expressed Genes in the Testis and Ovary of Mudskipper, *Boleophthalmus pectinirostris*

**DOI:** 10.3390/ani16010150

**Published:** 2026-01-05

**Authors:** He Ma, Chao Bian, Changxu Tian, Hongjuan Shi, Tianli Wu, Siping Deng, Guangli Li, Dongneng Jiang

**Affiliations:** 1 Fisheries College, Guangdong Ocean University, Zhanjiang 524088, China; 2 Guangdong Research Center on Reproductive Control and Breeding Technology of Indigenous Valuable Fish Species, Zhanjiang 524088, China; 3 Key Laboratory of Marine Ecology and Aquaculture Environment of Zhanjiang, Guangdong Ocean University, Zhanjiang 524088, China; 4 Guangdong Provincial Key Laboratory of Aquatic Animal Disease Control and Healthy Culture, Guangdong Ocean University, Zhanjiang 524088, China; 5 Laboratory of Aquatic Genomics, College of Life Sciences and Oceanography, Shenzhen University, Shenzhen 518057, China

**Keywords:** sex determination, sex differentiation, sex-biased genes, mudskipper

## Abstract

Sex determination and differentiation in teleosts are governed by complex genetic regulatory networks that include evolutionarily conserved mechanisms. In this study, we investigated *Boleophthalmus pectinirostris*, a Gobiidae species lacking heterogametic sex chromosomes, using comparative gonadal transcriptome analysis to identify sex differentially expressed genes (DEGs). RNA sequencing of ovarian and testicular tissues identified 17,214 DEGs, including 14,302 upregulated in males and 2912 upregulated in females. These DEGs were primarily associated with male (e.g., *dmrt1*, *amh*, *amhr2*) or female (e.g., *bmp15*, *gdf9*, *rspo1*) sex determination and differentiation, steroidogenesis (e.g., *hsd17b1*, *hsd3b1*, *cyp17a1*), and meiosis (e.g., *cyp26b1*, *aldh1a2*, *piwil2*). Functional enrichment analysis revealed that male upregulated DEGs were involved in spermatogenesis pathways such as calcium signaling, while female upregulated DEGs were associated with oogenesis pathways including oocyte meiosis and progesterone-mediated oocyte maturation. Conserved regulators, notably *dmrt1* and *amh*, were predicted to act as key hubs in protein–protein interaction networks, being primarily associated with reproductive processes and sex differentiation in *B. pectinirostris*. The *amh* gene produces two alternatively spliced isoforms that differ by a partial deletion in the second exon, both expressed in ovaries and testes. Collectively, this study provides the first comprehensive molecular framework of sex determination and differentiation in Gobiidae species, offering critical insights into the regulatory mechanisms of *B. pectinirostris* reproductive development.

## Simple Summary

Gobioidei fishes are key inhabitants of mangrove mudflats and intertidal zones, representing an important lineage in the transition from marine to terrestrial environments and occupying crucial ecological niches. They serve as essential food sources for many commercially important fishes, and some species, such as *Boleophthalmus pectinirostris*, also serve as a direct protein resources for humans. Gobioidei exhibit remarkable diversity and form one of the largest teleost groups, with over 2000 described species. However, morphological similarities among certain genera complicate accurate species and sex identification, underscoring the need for molecular tools. Here, we present the first transcriptome analysis of sex-biased gene expression in *B. pectinirostris*, an economically important species. We identified *amh*, a key gene in sex determination and differentiation, with two protein-coding isoforms. This study lays a foundation for understanding sex determination and differentiation in *B. pectinirostris* and advances insight into reproductive developmental mechanisms.

## 1. Introduction

The identification of sex-determining genes is a major focus in basic biology. Sex determination refers to the process by which an undifferentiated, bipotential gonad develops into either ovaries or testes [1,2]. In teleosts, sex determination is generally classified as genetic sex determination (GSD) or environmental sex determination (ESD). GSD is controlled by sex-determining genes located on sex chromosomes. These genes initiate cascades of sex-related signaling pathways and direct primordial gonads toward testis or ovary differentiation [3,4]. Teleost fish exhibit primitive, diverse, and highly plastic sex determination mechanisms [5,6]. Teleost fish sex chromosomes are often weakly differentiated, with small regions of recombination suppression and few heteromorphic features. This reflects the ancestral nature of teleost sex chromosome systems [4]. At the genomic level, frequent gene duplication, translocation, and sex chromosome turnover generate highly diverse sex-determining mechanisms, even among closely related species [5,6]. In addition, variation in water temperature, population density, and ecological stress can shift sex ratios, highlighting the high plasticity of sex determination in fish [1]. Together, these features make teleosts an ideal model for studying the evolution and functional diversification of vertebrate sex-regulatory networks.

Fish encompass almost all known sex-determining systems. Based on sex chromosome configurations, GSD in teleosts includes male heterogamety (XX/XY) and its variants, female heterogamety (ZZ/ZW) and its variants, as well as B chromosome (supernumerary chromosomes or extra chromosomes)-mediated sex determination [1]. In contrast to mammals and birds, which typically rely on a single, highly conserved sex-determining gene, fish possess multiple sex-determining or candidate genes [7]. These include members of the TGF-β (transforming growth factor-β) family and their downstream signaling components (e.g., *amh* and its Y-linked duplicate, the type II receptor *amhr2*, *gsdf*, *gdf6y*, and *bmpr1b*), Sox (Sry-related HMG-box) family genes (e.g., *sox3y*), DM-domain (doublesex and mab-3 related) genes (e.g., *dmrt1*), key steroidogenic enzymes (e.g., *hsd17b1*), and additional non-canonical regulators (e.g., *sdy*, *bcar1*, *irf9y*) [3,6]. These sex-determining genes are poorly conserved across teleosts and may differ even among congeneric species.

In contrast, genes involved in sex differentiation are relatively conserved. Male-biased regulators include *dmrt1*, *gsdf*, *sox9*, *amh*, and *amhr2*, whereas female-biased regulators include *foxl2*, *cyp19a1a*, *rspo1*, *sf1*, and *wnt4* [3,6]. These genes exert a central regulatory role in sex determination by regulating sex steroid synthesis. In teleosts, the principal female and male sex steroids are 17β-estradiol (E_2_) and 11-ketotestosterone (11-KT), respectively [2]. Notably, the terminal, rate-limiting steps of estrogen and androgen biosynthesis are completely separated in teleost. This differs from tetrapods, in which testosterone (T) is the precursor for E_2_ [8]. As a result, teleosts are a particularly suitable model for investigating how estrogens and androgens regulate sex determination and differentiation. During sex determination in fish, estrogens are indispensable, whereas androgens mainly act by counteracting estrogenic effects [2]. Sex-determining genes directly or indirectly regulate estrogen biosynthesis and thereby determine phenotypic sex [5]. Conversely, sex steroids also regulate the expression of sex-determining and differentiation genes. This feedback provides a mechanistic basis for hormone-induced sex reversal in fish [9]. Therefore, elucidating the regulatory mechanisms governing the expression of genes involved in sex determination and gonadal development is of fundamental importance.

Transcriptome analysis is a powerful tool for identifying sex-biased genes and has been widely applied in various teleost species. Common strategies include reference genome-based alignment (e.g., *Anguilla japonica*, *Siniperca chuatsi*, *Channa argus*, *Trachinotus blochii*, *Danio rerio*, *Larimichthys crocea*, *Takifugu rubripes*, *Centropyge vrolikii*, and *Trachidermus fasciatus*) and de novo assembly (e.g., *Oratosquilla oratoria*, *Siganus oramin*, *Macrobrachium rosenbergii*, and *Portunus trituberculatus*) to screen for sex-biased genes [10,11,12,13,14,15,16,17,18,19,20,21,22]. Differential expression analyses have revealed that in some teleost species, a greater number of male-upregulated genes are observed (e.g., *C. argus*, *T. rubripes*, *O. oratoria*, *L. crocea*, *T. fasciatus*, and *S. sihama*), whereas in others, female-upregulated genes predominate (e.g., *C. vrolikii*, *T. blochii*, and *D. rerio*) [10,11,12,13,14,15,16,17,18,19,20].

The mudskipper (*Boleophthalmus pectinirostris*) is an amphibious Gobiidae fish that is both a key species in mangrove and intertidal ecosystems and an important aquaculture species in Southeast Asia [23,24]. The female genome of *B. pectinirostris* has been sequenced, and karyotype analysis has revealed 23 pairs of chromosomes (2N = 46), but no sex chromosomes have been identified [25]. Artificial breeding of *B. pectinirostris* is still in its infancy. During the reproductive season, males are capable of multiple spermiations, whereas females spawn only once, with reproductive output directly limiting overall productivity [26]. Comparative transcriptomic analyses of gonadal tissues have not yet been reported for Gobiidae species. In this study, we characterized sex-biased DEGs in *B. pectinirostris* gonads through comparative transcriptomics, and identified DEGs associated with sex determination and differentiation, as well as the signaling pathways underlying steroidogenesis and gametogenesis. Furthermore, quantitative real-time PCR (qRT-PCR) was employed to validate the expression profiles of sex-related genes. Identifying sex-biased genes provides valuable insights into sex chromosome evolution, clarifies key regulators of sex differentiation in *B. pectinirostris*, and advances our understanding of reproductive development.

## 2. Materials and Methods

### 2.1. Animals and Sampling

Experimental fish were obtained from Xiashan Market (Zhanjiang, Guangdong, China). Fish were anesthetized with eugenol (20 mg/L, 5 min; Macklin, Shanghai, China) and gonadal tissues were dissected. A portion of tissue was fixed in Bouin’s solution (Phygene Biotechnology, Fuzhou, China) for histology, while the remainder was flash-frozen in liquid nitrogen for transcriptome sequencing. Female fish ranged from 14.7–15.6 cm in body length, weighed 24.31–27.67 g, and exhibited a gonadosomatic index (GSI) of 0.24–0.44%. Male fish ranged from 14.9–15.6 cm in body length, weighed 24.11–29.08 g, and had a GSI of 0.05–0.22%. All samples were collected in October, during the non-breeding season, when the gonads were in a post-reproductive state in two-year-old fish. Gonadal tissues from four randomly selected individuals per group were pooled as one biological replicate, with three replicates per sex. All procedures were approved by the Animal Ethics Committee of Guangdong Ocean University (no. 201903004).

### 2.2. Gonadal Histology

Fixed tissues were dehydrated through an ethanol gradient, cleared in xylene, paraffin-embedded, and sectioned at 5 μm thickness. Hematoxylin-eosin (H&E) staining was performed following previous protocols [24], with histological imaging conducted using a Leica DM500 microscope (Leica, Wetzlar, Germany) equipped with a digital camera system (Leica MC190 HD; Leica, Wetzlar, Germany).

### 2.3. RNA-Seq

Total RNA was isolated from gonadal tissues using TRIzol reagent (Invitrogen, Carlsbad, CA, USA) [24]. RNA purity (A260/A280 = 1.8–2.0) and integrity (28S:18S rRNA ratio close to 2:1) were verified using a Nanophotometer (Thermo Fisher, Waltham, MA, USA), a Qubit 2.0 fluorometer (Invitrogen, USA), and an Agilent 2100 Bioanalyzer (Agilent Technologies, Santa Clara, CA, USA). Qualified RNA samples were stored at −80 °C.

mRNA was enriched using Oligo (dT) magnetic beads, fragmented by ultrasonication, and reverse transcribed into double-stranded cDNA. Following end repair, adapter ligation, and size selection (~200 bp), libraries were amplified and sequenced on the Illumina NovaSeq 6000 platform (Illumina, San Diego, CA, USA; 150 bp paired-end reads) at Gene Denovo Biotechnology Co., Ltd. (Guangzhou, China).

Raw reads were processed with fastp (v0.18.0) to remove adapters, low-quality bases (Q ≤ 20), and rRNA contaminants (Bowtie2 v2.2.8). Clean reads were aligned to (HISAT2 v2.2.1) the *B. pectinirostris* reference genome (GCF_026225935.1) for subsequent analysis.

Gene expression levels were quantified as Fragments Per Kilobase of exon per Million mapped reads (FPKM) using StringTie (v1.3.1) and RSEM (v1.3.3). DEGs were identified with DESeq2 (v1.1.0; |log2FC| ≥ 1, FDR ≤ 0.05) between testis (T1–T3) and ovary (O1–O3) groups. DEGs were functionally annotated by GO and KEGG pathway enrichment. Novel genes, defined as those identified in the sequencing data but absent from the reference genome, were annotated using the NR, SwissProt, KEGG, and COG/KOG databases.

### 2.4. In Silico Analysis of Target Genes

Transcription factor binding motifs were retrieved from the JASPAR database (http://jaspar.genereg.net/ (accessed on 23 December 2024)). Target genes were predicted using MEME FIMO (v5.3.0), and regulatory networks were visualized in Cytoscape 3.7.1.

PPI networks were constructed using the STRING database (www.string-db.org (accessed on 23 December 2024)) and visualized in Cytoscape. Protein sequences from reference species were inferred by Blastx alignment.

Protein sequences of Dmrt1-6, Foxl2-3, and Amh were retrieved from the NCBI database and aligned using MAFFT (https://mafft.cbrc.jp/alignment/server/ (accessed on 23 July 2025)). Maximum-likelihood phylogenetic trees were constructed using the IQ-TREE web server with 1000 ultrafast bootstrap replicates (http://iqtree.cibiv.univie.ac.at/ (accessed on 23 July 2025)). Phylogenetic trees were visualized using FigTree (https://tree.bio.ed.ac.uk/software/figtree/ (accessed on 23 July 2025)). The aligned sequences used for phylogenetic analysis are provided in Table S7. Gene structure and synteny analyses were performed based on annotations from the NCBI database. Protein domain architectures were predicted using SMART (https://smart.embl.de/ (accessed on 23 July 2025)). cDNA from testis and ovary (diluted 10-fold) was used as the template for PCR amplification (35 cycles). Agarose gel electrophoresis revealed two distinct bands. The bands were excised separately, cloned into the pMD19-T vector (Takara, Dalian, China), and subjected to Sanger sequencing. The excised bands were purified individually and sequenced. Sequence chromatograms were analyzed using the Multi. ab1 Align function in TBtools (v2.39). Multiple sequence alignments were visualized with ESPript (https://espript.ibcp.fr/ESPript/cgi-bin/ESPript.cgi (accessed on 23 July 2025)).

### 2.5. Quantitative Real-Time PCR (qRT-PCR)

cDNA was synthesized 1 μg of total RNA (PrimeScript RT Master Mix, Takara, Dalian, China). The primers (Table S3) were designed using Primer-BLAST (NCBI, v2.5.0) and validated for amplification efficiency. qPCR was performed on a qTOWER2.2 system (Analytik Jena, Jena, Germany) with three reference genes (elongation factor 1α: XM_020932525.2, β-2-microglobulin: XM_020938238.2, β-actin: XM_020927288.2). Relative expression levels were calculated using the 2^−ΔΔCt^ method based on six biological replicates [23].

### 2.6. Statistical Analysis

Data are presented as mean ± SEM, with individual data points shown to better illustrate data distribution. Statistical analyses were performed using GraphPad Prism software (v10.3.1; GraphPad Software, San Diego, CA, USA). Comparisons between two groups of continuous variables were conducted using unpaired two-tailed Student’s *t*-tests (*p* < 0.05). When the F test indicated significant heterogeneity of variances, Welch’s corrected Student’s *t*-test was applied.

## 3. Results

### 3.1. Histological Characteristics of the Gonads

**Figure 1:**
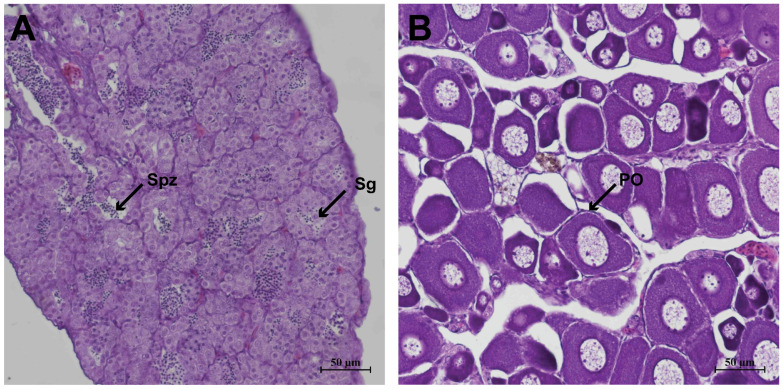
Histological characterization of gonadal tissues in *B. pectinirostris*. (**A**) Testicular tissue showing spermatogonia (Sg) and spermatozoa (Spz). (**B**) Ovarian tissue showing primary oocytes (PO).

Histological observation showed that the testes predominantly contained spermatogonia, with rare meiotic cells, and some luminal spermatozoa. In contrast, the ovaries contained numerous primary oocytes surrounded by a single layer of follicular cells with predominantly euchromatic nuclei (Figure 1).

### 3.2. Transcriptome Data Quality and Identification of DEGs

**Figure 2:**
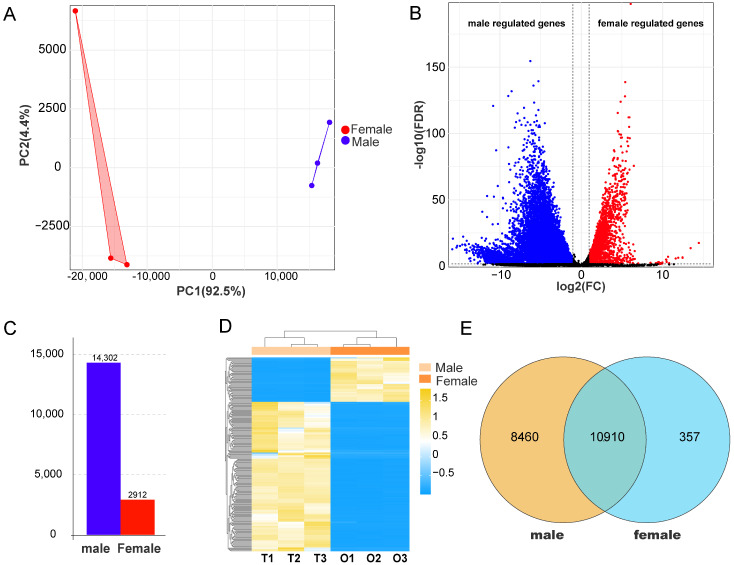
Transcriptomic divergence between testicular and ovarian tissues. (**A**) Principal component analysis separates male (blue dots) and female (red dots) samples. (**B**) Volcano plot shows DEGs, with male/female upregulated DEGs (|log2FC| ≥ 1, FDR ≤ 0.05, dashed lines indicate thresholds). (**C**) Bar chart shows the number of sex-biased DEGs. (**D**) Hierarchical clustering of the top 200 DEGs. (**E**) Venn diagram shows sex-specific genes.

RNA-seq results showed that each sample generated about 6.2 GB of data. All six gonadal cDNA libraries demonstrated high sequencing quality, with Q20 ≥ 97.66%, Q30 ≥ 93.32%, GC content ≥ 46.39%, and exon mapping rate ≥ 88.13% (Table S1), confirming the reliability of downstream analyses. Principal component analysis (PCA) revealed clear transcriptomic separation between ovarian and testicular samples (Figure 2A).

A total of 27,142 genes were identified, including 22,865 annotated genes and 4277 novel genes. Testicular tissues expressed 21,708–21,820 genes, while ovarian tissues expressed 18,260–18,634 genes (Table S2). Volcano and bar plots revealed 17,214 DEGs, comprising 14,302 upregulated in males and 2912 upregulated in females (Figure 2B,C). Hierarchical clustering of the top 200 DEGs produced sex-specific expression patterns, corroborating the PCA results (Figure 2D). Additionally, 8460 male-specifically and 357 female-specifically expressed genes were detected (Figure 2E).

### 3.3. Functional Enrichment Analysis of DEGs

**Figure 3:**
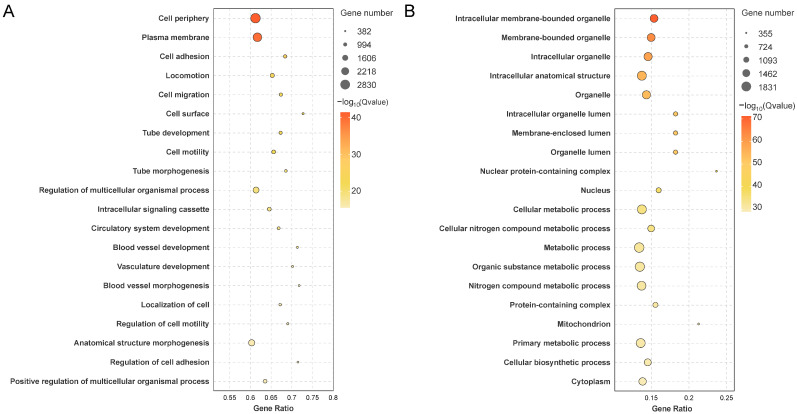
GO enrichment analysis of sex-biased DEGs. (**A**) GO terms enriched in male-upregulated DEGs. (**B**) GO terms enriched in female-upregulated DEGs. Terms are ranked by ascending Q-values.

GO analysis classified DEGs into biological processes, cellular components, and molecular functions (Figure S1). Overall, DEGs were mainly enriched in “catalytic activity” (GO:0003824), “circulatory system development” (GO:0072359), and “regulation of cell differentiation” (GO:0045595) (Figure S3A). Male-upregulated DEGs were enriched in “regulation of multicellular organismal process” (GO:0071345) and “circulatory system development” (GO:0001578) (Figure 3A), while female-upregulated DEGs were associated with “cellular metabolic process” (GO:0044237) and “organic substance biosynthetic process” (GO:0044249) (Figure 3B). Male-specific expression genes enriched in “cell periphery” (GO:0071944), “plasma membrane” (GO:0005886) (Figure S4A), while female-specific genes were enriched in “nucleosome” (GO:0000786), “FANCM-MHF complex” (GO:0071821), and “transcription factor TFIID complex” (GO:0005669) (Figure S4B).

**Figure 4:**
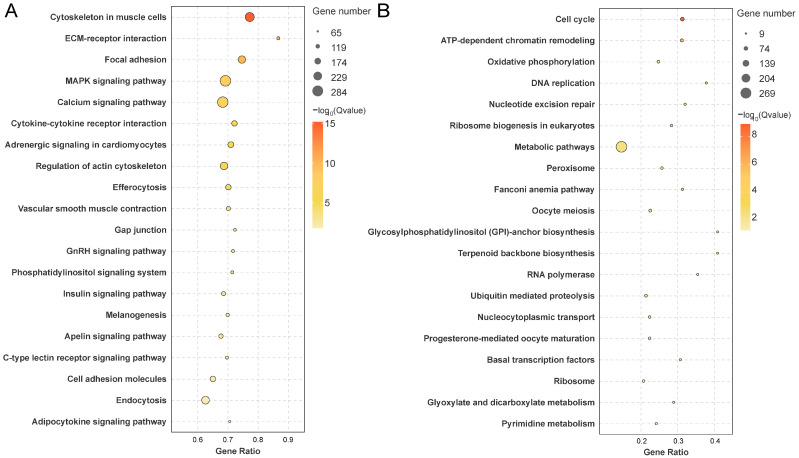
KEGG pathway enrichment analysis of sex-biased DEGs. (**A**) KEGG pathways enriched among male-upregulated DEGs. (**B**) KEGG pathways enriched among female-upregulated DEGs. Pathways are ranked by ascending Q-values.

KEGG pathway analysis classified DEGs into six categories: Metabolism, Human Diseases, Organismal Systems, Genetic Information Processing, Cellular Processes, and Environmental Information Processing (Figure S2). All DEGs enriched pathways included “ECM-receptor interaction” (ko04512), and “steroid hormone biosynthesis” (ko00140) (Figure S3B). Male-upregulated DEGs were associated with “MAPK signaling” (ko04010), “calcium signaling” (ko04020), and “GnRH signaling” (ko04912) (Figure 4A), while female-upregulated DEGs were enriched in “oocyte meiosis” (ko04114), “progesterone-mediated oocyte maturation” (ko04914), and “transcriptional regulation” (ko03022) (Figure 4B). Male-specific expression genes were enriched in “cytokine-cytokine receptor interaction” (ko04060), “calcium signaling pathway” (ko04020), and “steroid hormone biosynthesis” (ko06019) (Figure S5A), whereas female-specific expression genes were associated with “ATP-dependent chromatin remodeling” (ko03082), “necroptosis” (ko04217), and “cell adhesion molecules” (ko04514) (Figure S5B).

### 3.4. Expression of Representative DEGs

**Figure 5:**
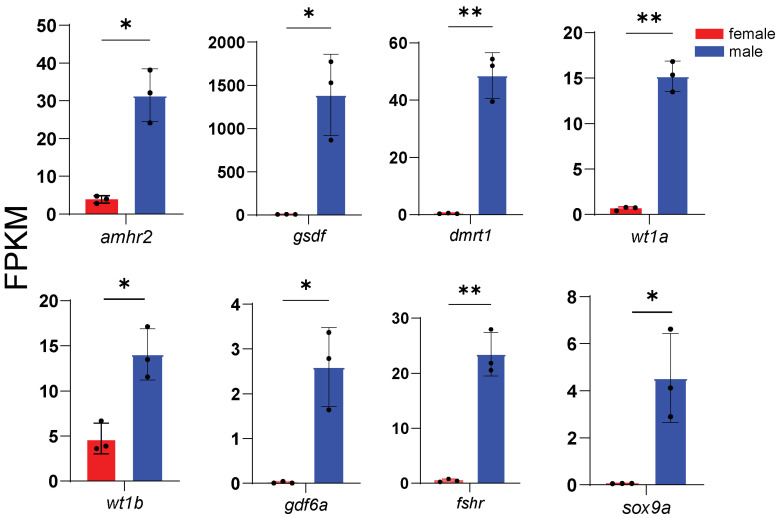
Histograms showing the expression of genes associated with male sex determination and differentiation. The data are based on the FPKM values obtained from the RNA-seq data. *, *p* < 0.05; **, *p* < 0.01.

**Figure 6:**
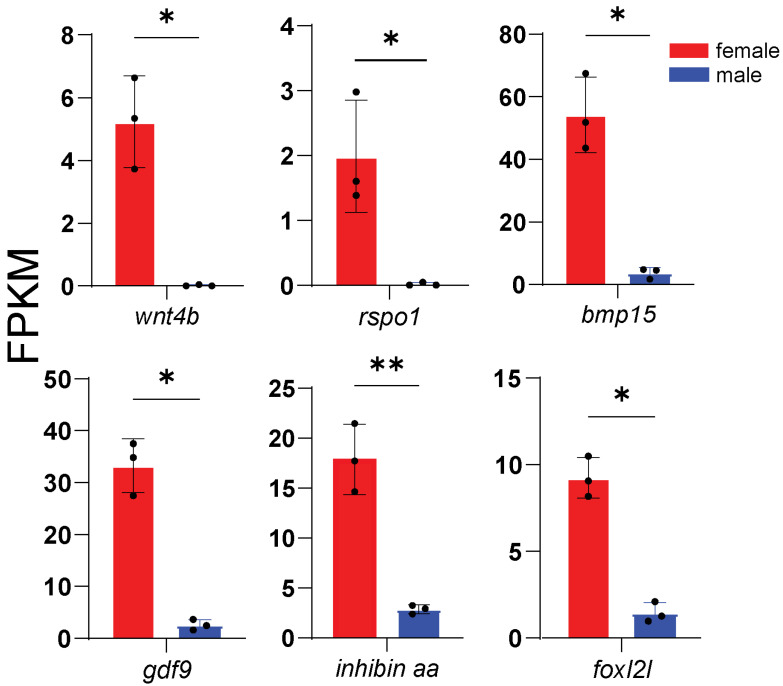
Histogram showing the expression of genes associated with female sex determination and differentiation. The data is based on the FPKM values obtained from the RNA-seq data. *, *p* < 0.05; **, *p* < 0.01.

**Figure 7:**
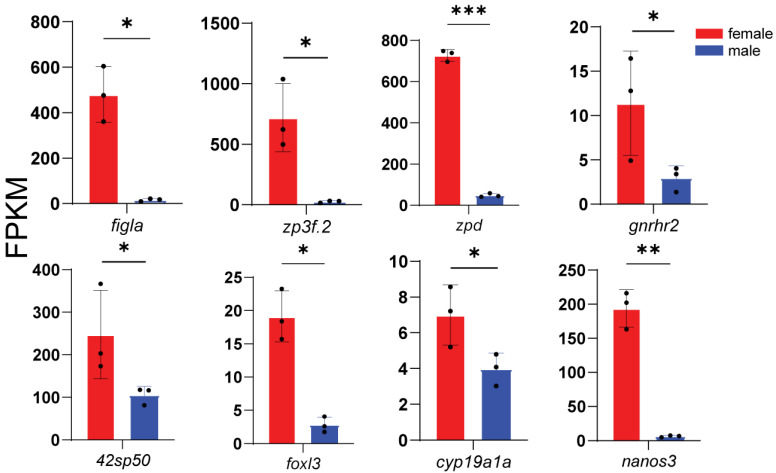
Histograms showing the expression of genes associated with oogenesis. The data are based on the FPKM values obtained from the RNA-seq data. *, *p* < 0.05; **, *p* < 0.01; ***, *p* < 0.001.

**Figure 8:**
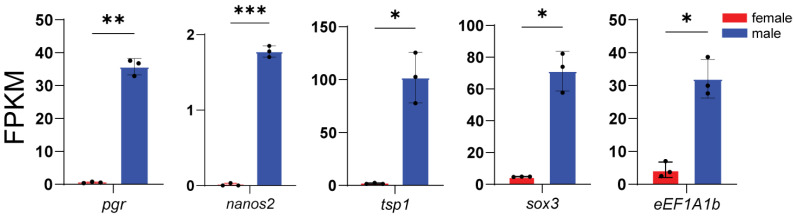
Histograms showing the expression of genes associated with spermatogenesis. The data are based on the FPKM values obtained from the RNA-seq data. *, *p* < 0.05; **, *p* < 0.01; ***, *p* < 0.001.

**Figure 9:**
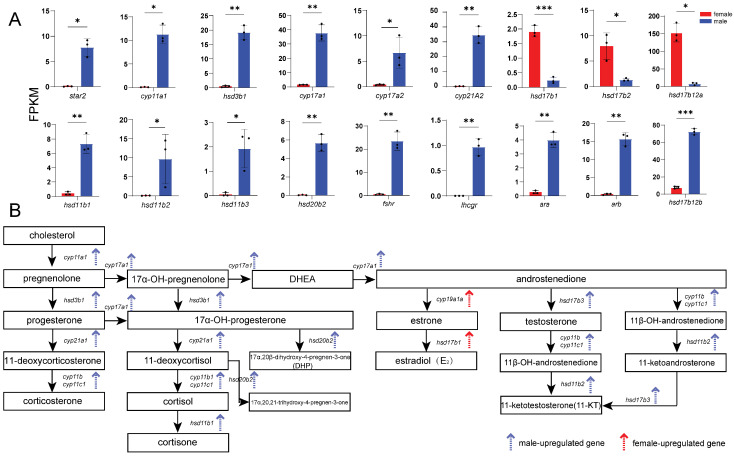
Histograms showing the expression of genes associated with steroidogenesis. (**A**) The data are based on the FPKM values obtained from the RNA-seq data. *, *p* < 0.05; **, *p* < 0.01; ***, *p* < 0.001. (**B**) Schematic representation of steroidogenic pathway in fish. Red arrows indicate genes upregulated in females, and blue arrows indicate genes upregulated in males.

**Figure 10:**
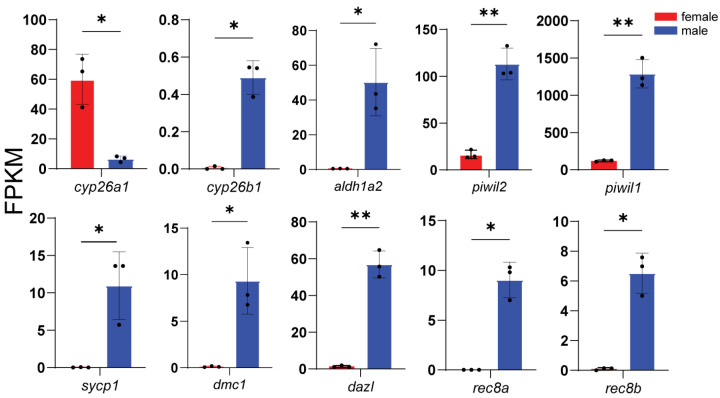
Histograms showing the expression of genes associated with meiosis. The data are based on the FPKM values obtained from the RNA-seq data. *, *p* < 0.05; **, *p* < 0.01.

According to enrichment analysis, we identified genes related to key processes: male sex determination and differentiation (*amhr2*, *gsdf*, *sox9a*, *wt1a*, *wt1b*, *gdf6a*, and *fshr*) (Figure 5), female sex determination and differentiation (*foxl2l*, *wnt4b*, *rspo1*, *bmp15*, *gdf9*, and *inhibin aa*) (Figure 6), oogenesis (*figla*, *foxl3*, *cyp19a1a*, *nanos3*, *lrh*, *zp3f.2*, *zpd*, *nr5a2*, and *gnrhr2*) (Figure 7), spermatogenesis (*pgr*, *nanos2*, *elof1*, *tsp1*, and *sox3*) (Figure 8), steroidogenesis (*sf1*, *hsd17b1*, *hsd17b2*, *hsd17b3*, *hsd17b12a*, *hsd17b12b*, *hsd11b1*, *hsd11b2*, *hsd11b3*, *hsd20b2*, *hsd3b1*, *cyp11a1*, *cyp21a2*, *cyp17a1*, *cyp17a2*, *star2*, *ara*, *arb*, *esr2a*, *esr1*, *fshr* and *lhcgr*) (Figure 9), and meiosis (*cyp26a1*, *cyp26b1*, *aldh1a2*, *piwil2*, *piwil1*, *sycp3*, *dmc1*, *dazl*, *rec8a*, and *rec8b*) (Figure 10). Interestingly, the female-upregulated DEGs were primarily those involved in the estrogen synthesis pathway (e.g., *hsd17b12a*, *hsd17b12b*), while the male-upregulated DEGs encompassed the majority of genes in the steroid hormone biosynthesis pathway (Table S4).

### 3.5. Core Sex-Related Genes and Regulatory Networks

**Figure 11:**
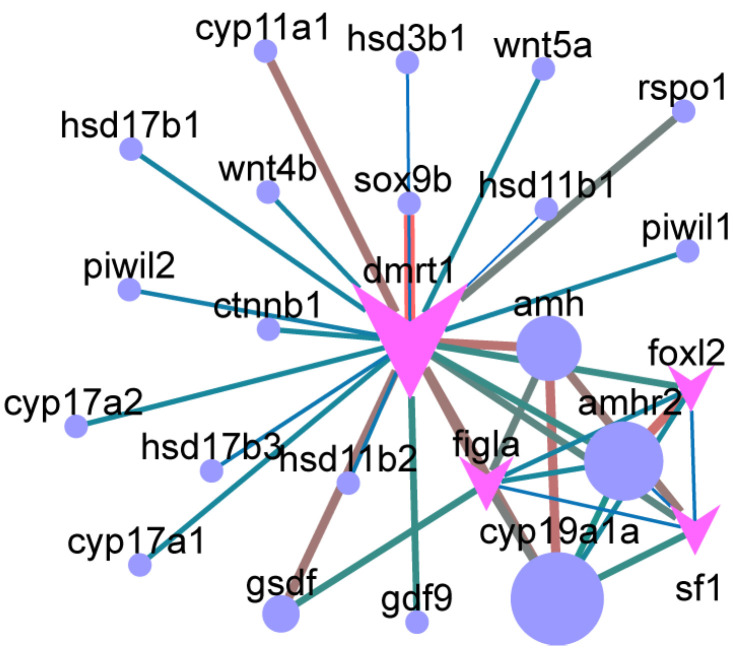
Protein–protein interaction (PPI) network of core regulators. Node size reflects degree (larger nodes indicate more connections), while edge thickness indicates interaction strength. Core genes (red arrows) are linked by blue circles, with circle size proportional to connection significance; larger circles represent greater importance.

PPI network analysis further revealed *dmrt1* and *amh* as central hubs in sex determination and differentiation pathways (Figure 11). PPI were predicted interactions inferred from orthology-based databases, rather than experimentally validated physical interactions in *B. pectinirostris*. *amh* was connected to male-biased genes such as *amhr2*, *dmrt1*, and *sf1*, as well as female-biased genes including *foxl2*, *cyp19a1a*, and *figla*. Similarly, *dmrt1* exhibited interactions with male-biased genes (*amh*, *gsdf*, *sox9b*, *amhr2*) and female-biased genes (*foxl2*, *gdf9*, *figla*, *cyp19a1a*, and *rspo1*). Chromosomal mapping of sex-biased differentially expressed genes revealed that many are located on chromosomes 4 (*sf1*, *piwil2*, *piwil1*, *dmrt2a*, *dmrt3a*, *dmrt1*, *hsd17b3*) and 7 (*dmrt2b*, *lrh*, *dmrt5*, *amh*, *hsd11b1*) (Figure S6).

### 3.6. Key Gene Family in Silico Analysis

**Figure 12:**
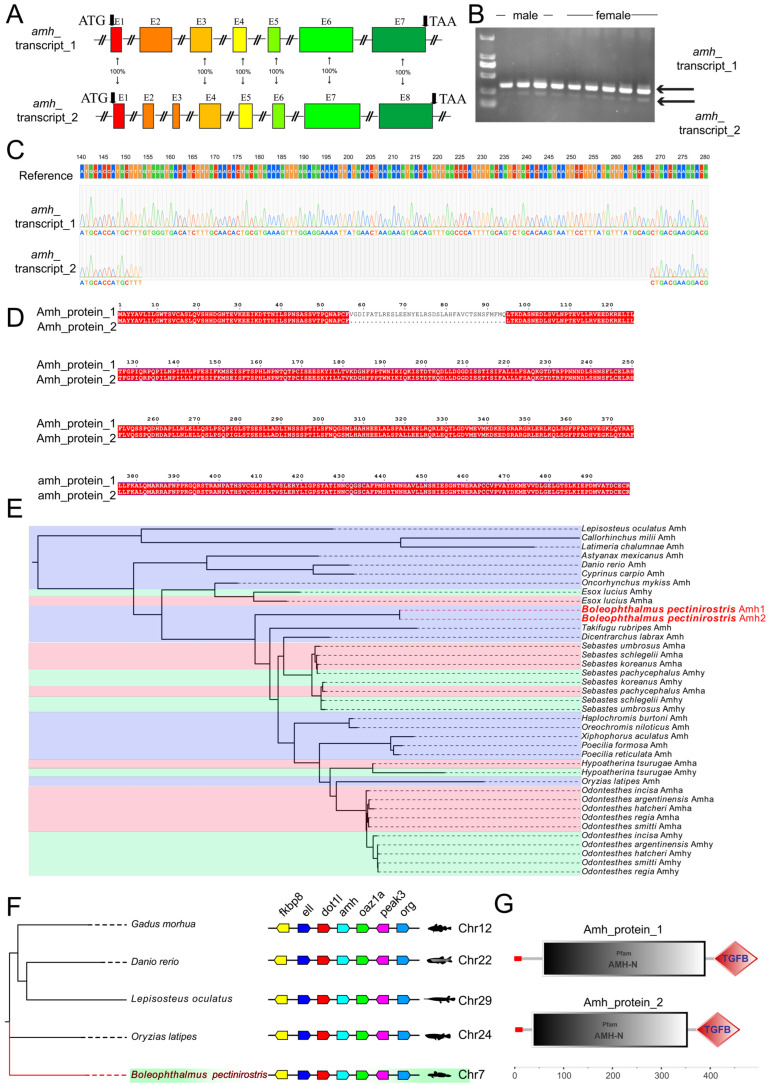
Phylogenetic and genomic characterization of *amh* gene family in vertebrates. (**A**) Gene structures of *amh* in *B. pectinirostris* display exon-intron organization and alternative splicing patterns. (**B**) RT-PCR amplification of alternative splicing exon region. (**C**) Sequencing chromatograms of the alternatively splicing exon region. (**D**) Alignment of Amh protein sequence. (**E**) A maximum likelihood phylogenetic tree reveals evolutionary relationships of Amh proteins across vertebrate lineages. (**F**) Synteny analysis shows conserved genomic organization of the *amh* locus across representative teleosts. (**G**) Amh proteins in *B. pectinirostris* contain conserved functional domains.

Further analyses were conducted on the gene families involved in key male (*amh* and *dmrt1*) and female (*foxl2* and *foxl3*) sex determination and differentiation. In *B. pectinirostris*, a single *amh* gene was detected, which exhibits two alternatively spliced isoforms differing by a partial coding sequence deletion within the second exon (Figure 12A). Amplification of the differential fragment of the second exon from male and female cDNA yielded two bands (Figure 12B). The sequencing chromatograms revealed a difference of 114 bp between the two amplification products (Figure 12C). Analysis of the amino acid sequences indicated that they encode 499 and 461 amino acids, respectively, with no frameshift mutation observed (Figure 12D). Phylogenetic analysis revealed the presence of both *amhy* (Y-chromosome copy) and *amha* (autosomal copy) genes in multiple teleost species (Figure 12E). The *amh* gene harbors conserved AMH and TGF-β domains (Figure 12F), and synteny analysis revealed highly conserved flanking gene arrangements (Figure 12G).

Transcriptome data further identified six *dmrt* family members in *B. pectinirostris* (Figure S7A), each comprising 2–5 exons (Figure S7B). All DMRT proteins share a conserved DM domain located at the N-terminus (Figure S7C). Additionally, a relatively conserved *dmrt1*-*dmrt3*-*dmrt2a* gene cluster was found, with flanking genes arranged in a conserved syntenic pattern (Figure S7D).

Gene family analysis showed that the *B. pectinirostris* possesses three genes, *foxl2*, *foxl2l*, and *foxl3*, each forming an independent clade (Figure S8A). In some teleost species, *foxl2* has undergone duplication, giving rise to *foxl2a* and *foxl2b*, with *foxl2b* forming a distinct clade and *foxl2a* clustering with the single *foxl2* present in most teleosts. Structural analysis indicated that *B. pectinirostris foxl2*, *foxl2l*, and *foxl3* contain 1, 3, and 4 exons (Figure S8B), respectively, all harboring a conserved forkhead DNA-binding domain (Figure S8C), and that the upstream and downstream regions of *foxl2*, the downstream region of *foxl2l*, and the upstream region of *foxl3* are relatively conserved (Figure S8D).

### 3.7. Transcriptome Data Validation

**Figure 13:**
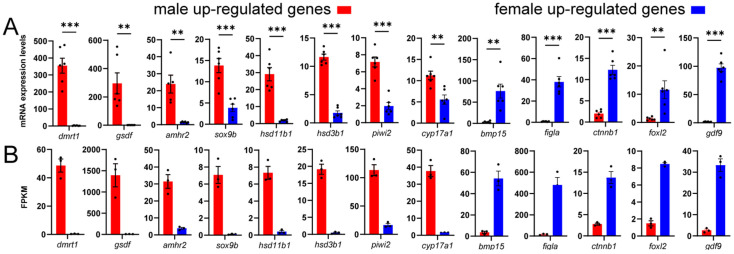
Validation of RNA-seq data by qRT-PCR. (**A**) Relative expression levels of selected sex-related DEGs determined by qRT-PCR. The *Y*-axis indicates relative transcription levels. Student’s *t*-test determines statistical significance (** represents *p* < 0.01, *** represents *p* < 0.001). (**B**) FPKM values from RNA-seq showing consistent expression trends for the same genes.

Eighteen genes were randomly selected for quantitative validation (Figure 13A). Despite differences in fold changes, similar expression trends were observed between FPKM values of transcriptome data and relative expression levels measured by qRT-PCR (Figure 13B).

## 4. Discussion

In this study, comparative transcriptomic analysis of testes and ovaries in *B. pectinirostris* identified 17,214 sex-biased DEGs, including 14,302 upregulated in males and 2912 upregulated in females, as well as 8460 male-specific and 357 female-specific genes. Many of these DEGs were associated with known sex determination and differentiation pathways (e.g., *dmrt1* and *amh*), and with gametogenesis and steroidogenesis. Together, these results provide a comprehensive framework for understanding gonadal development in this species

In this study, male upregulated genes substantially outnumbered female upregulated genes, a pattern also observed in multiple teleost species [10,11,12,13,14,15,16,17,18,19,20]. This disparity likely reflects fundamental biological differences in gonadal structure and reproductive function [27,28]. In *B. pectinirostris*, males possess seminal vesicle for sperm storage that relies on sustained testicular support. In addition, sampling was conducted outside the reproductive season, when ovaries were at the oocyte growth stage, a phase associated with elevated RNase activity that may reduce RNA integrity and transcript detection sensitivity. Together, these biological and tissue-specific factors likely contribute to the predominance of male upregulated genes observed in this study.

### 4.1. Two Alternatively Spliced Transcripts of amh Are Expressed in Both Testes and Ovaries

In teleosts, *amh*, *amha*, and *amhy* with conserved TGF-β domains have been identified [29]. *amh* is broadly expressed in gonadal somatic cells and shows higher expression in male than in female gonads during development [30,31]. Among 114 teleost species examined, sex-determining genes in 75 species belong to the TGF-β superfamily, including *amh*, *amhr2*, *gsdf*, *gdf6*, *bmpr1b*, and *id2b* [32]. Specifically, *amh* has been identified as the sex-determining gene in 33 species from 12 genera, such as *Oryzias eversi*, whereas the Y chromosome-specific duplicate *amhy* is the sex-determining gene in *O. niloticus* and *Odontesthes hatcheri* [30]. In addition, the type II receptor *amhr2* acts as the sex-determining gene in *T. rubripes*, *Silurus lanzhouensis*, *Silurus meridionalis*, and *Perca flavescens* [33,34,35,36,37,38,39,40]. Thus, TGF-β superfamily members, especially *amh* and *amhr2*, account for a large fraction of known teleost sex-determining genes, and the TGF-β pathway is unique in that both ligands and receptors participate directly in sex determination. However, owing to the plasticity and diversity of sex determination and differentiation mechanisms in teleosts, the function of *amh* varies considerably among species [4]. For example, in zebrafish, the loss of *amhr2* leads to *amh* signaling through the type II receptor *bmpr2a*, thereby promoting male sex differentiation and spermatogenesis [31,41]. Here, we identified two alternatively spliced *amh* transcripts in *B. pectinirostris*, expressed in both testes and ovaries. The isoforms differ by a 114 bp fragment and encode proteins of 499 and 461 amino acids, respectively, with identical domain organization, suggesting similar functions. Both variants are expressed in females, indicating that *amh* may directly regulate oogonial proliferation and differentiation through paracrine signaling, or indirectly influence female germ cell development via interactions between somatic and germ cells [42]. Furthermore, PPI analysis revealed a potential regulatory relationship between *amh* and *cyp19a1a*, implying that Amh may act through the classical SMAD signaling cascade or via the Rora/Ncoa5 pathway to regulate *cyp19a1a* expression [9], a mechanism that warrants further investigation.

### 4.2. Enrichment of Sex-Determining and Differentiation Genes

We identified multiple conserved sex-related genes that were significantly enriched in the PPI network. Among them, *dmrt1* occupied a central hub position. *dmrt1* is highly expressed in both germ and somatic cells of the teleost testis. It is typically expressed early in undifferentiated gonads and exhibits pronounced sexual dimorphism during gonadal differentiation, with significantly higher or male-specific expression in testes and minimal or undetectable expression in ovaries and other somatic tissues [43]. *dmrt1* functions as a key regulator of testis differentiation, maintenance of the male phenotype, and germ cell development [44,45,46]. *dmrt1* has been identified as a major or critical sex-determining gene in chickens, one amphibian species, one fish species, and one turtle species, and has been proposed as a candidate sex-determining gene in an additional 12 species (including six fish, two amphibians, and four birds) [43]. *dmrt1* directly regulates the transcription of *gsdf* [46]. Here, six members of the *dmrt* gene family were differentially expressed. However, no male-specific copy of *dmrt1* was detected in *B. pectinirostris*. In contrast, male-specific *dmrt1* copies have been reported in Scatophagidae species, where they may arise through gene duplication or allelic variation and act as sex-determining factors [47]. These results suggest that *dmrt1* in *B. pectinirostris* is more likely involved in sex differentiation rather than serving as an upstream sex-determining gene.

*gsdf* is primarily expressed in ovarian granulosa cells and testicular Sertoli cells. It plays essential roles in gonadal differentiation and germ cell proliferation in teleosts [48,49,50]. In several Sebastes species, the Y-linked copy *gsdfy* has been identified as the master sex-determining gene [51]. Mechanistically, *gsdf* transcription is directly activated by Dmrt1 and Sf1. Gsdf protein can also interacts with Ncoa5 to suppress Rora/Ncoa5-mediated *cyp19a1a* transcription [9]. In *B. pectinirostris*, *gsdf*, *sf1*, and *rora* were all upregulated in males. This expression pattern suggests that the regulatory relationship among these genes is conserved. Together, these findings support an important role for *gsdf* in male differentiation in this species.

Foxl2 is a key transcription factor maintaining the female phenotype, and is predominantly expressed in teleost fish somatic cells [52,53]. *foxl2* promotes ovarian differentiation and oogenesis by directly regulating *cyp19a1a* [52]. In *B. pectinirostris*, *foxl2* showed strong predicted interactions with *amh* and *cyp19a1a* in the PPI network, indicating a central role in ovarian differentiation.

The oocyte-related genes *gdf9*, *bmp15*, and *figla* were also highly expressed in females [54,55,56]. Both *gdf9* and *bmp15* belong to the TGF-β superfamily and often form heterodimers to activate BMP receptors and downstream SMAD signaling, thereby regulating folliculogenesis and oocyte maturation [48,57,58,59]. *figla* contributes to early follicle formation and maintenance of ovarian development via estrogen signaling [54,60]. Their upregulation in females in this study indicates that these genes are likely essential for ovarian development in *B. pectinirostris*. In cichlid hybrids utilizing the LG1 sex determination system, the *figla-like* gene has been identified as the key regulator of male sex determination [61]. In the present study, only a single *figla* gene was detected, which was upregulated in females; however, its functional role remains to be elucidated. Unlike the continuous spermatogenesis observed in males, ovarian development is characterized by stage-specific oocyte growth and maturation, which may explain the more restricted and temporally specific transcriptional activation observed in females and the relatively smaller number of female-upregulated genes.

### 4.3. Differentially Expressed Genes Involved in Gametogenesis and Steroidogenesis

Teleost gametogenesis comprises three main stages: germ cell proliferation, meiotic division, and gamete maturation. These processes are tightly regulated by sex steroids [27,28]. Steroid biosynthesis begins with cholesterol transport into mitochondria by *StAR*. Cholesterol is then converted into pregnenolone by *cyp11a1* [62]. Subsequent steps catalyzed by *hsd3b1* and *cyp17a1* lead to the production of testosterone, which is finally converted into 11-ketotestosterone by *cyp11c1* [63,64]. In *B. pectinirostris*, these steroidogenic genes were significantly upregulated in male gonads, consistent with histological observations, highlighting the essential role of androgens in spermatogenesis and male differentiation.

GO enrichment analysis revealed significant overrepresentation of germ cell development and meiotic cell cycle. Combined with male-biased upregulation of genes involved in steroid biosynthesis (e.g., *star*, *cyp11a1*, *hsd3b1*, *cyp17a1*, and *cyp17a2*) and receptor signaling (e.g., *arα* and *arβ*), these results indicate that the transcriptome captures a largely complete spermatogenic program, spanning meiotic progression (e.g., *sycp3*, *dax1*, *spo11*, *dmc1*, and *rec8*) and sperm formation (e.g., *spata7*), consistent with histological observations. In addition, key receptors and transcription factors involved in spermatogenesis and reproductive axis regulation (e.g., *sox9*, *wt1*, *sf1*, *fshr*, and *lhr*) were upregulated in males, supporting an integrated regulatory framework linking endocrine signaling, local paracrine regulation, and nuclear receptor-mediated transcription. These findings highlight coordinated activation of the “steroidogenesis–receptor signaling–gametogenesis” axis in males, reflecting sustained transcriptional activity required for continuous sperm production and maintenance of steroidogenic and supportive somatic cell functions [2].

We also found that the nuclear receptor for 17α,20β-dihydroxy-4-pregnen-3-one (DHP, the major progestin in most teleosts), *pgr*, was significantly upregulated in males. DHP is traditionally considered a female associated progestin, yet it also participates in male reproduction [2,8]. DHP play critical roles in spermatogenesis, sperm maturation and spermiation partially through activating Pgr in most teleost [2,65]. In addition, regarding estrogen biosynthesis, testosterone is converted into estrogens via *hsd17b1* and *cyp19a1a*. Both genes were highly expressed in females, which is consistent with their roles in ovarian steroidogenesis and oocyte development.

Meiosis-associated genes *dax1* and *sycp3* were also specifically upregulated in males [66,67]. Loss-of-function studies in zebrafish have shown that disruption of either gene results in female-to-male sex reversal [68,69]. These findings suggest that *dax1* and *sycp3* are predominantly involved in male germ cell development in *B. pectinirostris*, rather than serving as key regulators of female sex determination.

**Figure 14:**
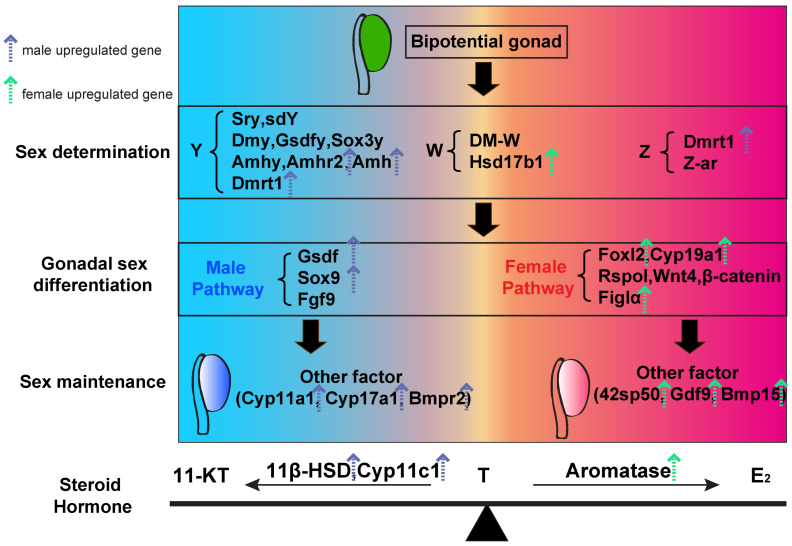
Integrated model of gonadal development regulation in teleost fish. The bipotential gonad diverges into testis (left, blue) or ovary (right, red) through genetic cascades and steroid hormone interactions. Male-related and female-related genes are marked in blue and red, respectively. Key hormones include 11-KT, T, and E_2_. Blue and green arrows represent the up-regulated genes in male and female *B. pectinirostris*, respectively.

Sex determination and differentiation in teleosts can be broadly divided into three hierarchical levels: upstream sex determination, midstream sex differentiation networks, and downstream gametogenesis and steroidogenesis [1]. Upstream sex-determining factors are activated in undifferentiated gonads. These factors direct development toward the male (e.g., *dmrt1*, *amh*, *amhr2*, *gsdf*) or the female (e.g., *foxl2*) pathway. This regulation may involve a single master gene or the combined effects of multiple genes and/or environmental cues. Upstream signals are integrated by transcription factors (e.g., male: *sox9a*/*sox9b*, *wt1*; female: *foxl2*, *foxl3*, *figla*, *nobox*), TGF-β family members (e.g., male: *amh*, *amhr2*, *gsdf*, *gdf6*; female: *amh*, *gsdf*, *gdf9*, *bmp15*), and nuclear receptors (e.g., male: *sf1*; female: *lrh1*) to establish sex-specific gene expression networks that regulate gonadal cord formation, the fate of Sertoli cells and germ cells, and steroidogenic pathways during sex differentiation. Downstream, gametogenesis and steroidogenesis drive sperm and oocyte production and, together with the dynamic regulation of androgen, estrogen, and progestin levels, result in the formation of functional gonads. These processes are ultimately coordinated by the hypothalamus–pituitary–gonadal (HPG) axis, specifically the GnRH–GtH (Fsh/Lh)–gonadal steroid cascade (Figure 14).

## 5. Conclusions

This study systematically compared ovarian and testicular transcriptomes of *B. pectinirostris.* Critical genes and pathways involved in sex determination, differentiation, gametogenesis, and steroidogenesis were identified, including alternatively spliced variants of *amh*. The findings deepen the understanding of the evolutionary origin and plasticity of sex determination mechanisms in *B. pectinirostris* and provide a valuable resource for future studies on reproductive biology in Gobiidae fishes.

## Data Availability

All sequencing data from clean libraries were submitted to the China National Center for Bioinformation Genome Sequence Archive with a Bioproject number: CRA035924. Available online: https://ngdc.cncb.ac.cn/gsa/s/W84r20yz (accessed on 30 December 2025).

## Supplementary Materials

The following supporting information can be downloaded at: https://www.mdpi.com/article/10.3390/ani16010150/s1, Figure S1: GO functional classification of all sex-biased DEGs; Figure S2: KEGG functional classification of all sex-biased DEGs; Figure S3: Pathway enrichment analysis of all sex-biased DEGs; Figure S4: GO enrichment analysis of sex-specific expression genes. Figure S5: KEGG enrichment analysis of sex-specific expression genes. Figure S6: Chromosomal distribution of sex determination and differentiation genes. Figure S7: Phylogenetic and genomic characterization of *dmrt* gene family in vertebrates. Figure S8: Phylogenetic and genomic characterization of *foxl2* and *foxl3* gene family in vertebrates. Table S1: Summary of RNA-seq quality metrics; Table S2: Summary of gene expression statistics; Table S3: Primers used for the gene expression analysis; Table S4: Functional annotation of differentially expressed sex-related genes; Table S5: All expressed genes identified in female and male gonads; Table S6: Genes upregulated in female and male gonads; Table S7: Protein sequences used for phylogenetic tree construction.
