# Supplementary material for: Comparative RNA-Seq Analysis of Differentially Expressed Genes in the Testis and Ovary of Mudskipper, Boleophthalmus pectinirostris
Source: Animals (Basel). 2026 Jan 5;16(1):150. doi: 10.3390/ani16010150 (PMC12784797; doi:10.3390/ani16010150)
Supplement: Supplementary file 1 [file animals-16-00150-s001.zip › Gene abbreviation .docx]

| **Gene symbol** | **Full gene name** |
| --- | --- |
| ***aldh1a2*** | Aldehyde dehydrogenase 1 family member A2 |
| ***amh*** | Anti-Müllerian hormone |
| ***amhr2*** | Anti-Müllerian hormone receptor type 2 |
| ***ara*** | Androgen receptor alpha |
| ***arb*** | Androgen receptor beta |
| ***bcar1*** | Breast cancer anti-estrogen resistance protein 1 |
| ***bmp15*** | Bone morphogenetic protein 15 |
| ***bmpr1*** | Bone morphogenetic protein receptor type 1 |
| ***bmpr2*** | Bone morphogenetic protein receptor type 2 |
| ***cyp11a1*** | Cytochrome P450 family 11 subfamily A member 1 |
| ***cyp17a1*** | Cytochrome P450 family 17 subfamily A member 1 |
| ***cyp17a2*** | Cytochrome P450 family 17 subfamily A member 2 |
| ***cyp19a1a*** | Cytochrome P450 family 19 subfamily A member 1a (aromatase) |
| ***cyp21a2*** | Cytochrome P450 family 21 subfamily A member 2 |
| ***cyp26a1*** | Cytochrome P450 family 26 subfamily A member 1 |
| ***cyp26b1*** | Cytochrome P450 family 26 subfamily B member 1 |
| ***dax1 (nr0b1)*** | Dosage-sensitive sex reversal, adrenal hypoplasia critical region, on chromosome X, gene 1 |
| ***dazl*** | Deleted in azoospermia-like |
| ***dmrt1*** | Doublesex and mab-3 related transcription factor 1 |
| ***dmc1*** | DNA meiotic recombinase 1 |
| ***elof1*** | Elongation factor 1 homolog |
| ***esr1*** | Estrogen receptor 1 |
| ***esr2a*** | Estrogen receptor 2a |
| ***figla*** | Factor in the germline alpha |
| ***foxl2*** | Forkhead box L2 |
| ***foxl2l*** | Forkhead box L2-like |
| ***foxl3*** | Forkhead box L3 |
| ***fshr*** | Follicle-stimulating hormone receptor |
| ***gdf6y*** | Growth differentiation factor 6, Y-linked |
| ***gdf9*** | Growth differentiation factor 9 |
| ***gnrhr2*** | Gonadotropin-releasing hormone receptor 2 |
| ***gsdf*** | Gonadal soma-derived factor |
| ***hsd11b1*** | Hydroxysteroid 11-beta dehydrogenase 1 |
| ***hsd11b2*** | Hydroxysteroid 11-beta dehydrogenase 2 |
| ***hsd11b3*** | Hydroxysteroid 11-beta dehydrogenase 3 |
| ***hsd17b1*** | Hydroxysteroid 17-beta dehydrogenase 1 |
| ***hsd17b2*** | Hydroxysteroid 17-beta dehydrogenase 2 |
| ***hsd17b3*** | Hydroxysteroid 17-beta dehydrogenase 3 |
| ***hsd17b12a*** | Hydroxysteroid 17-beta dehydrogenase 12a |
| ***hsd17b12b*** | Hydroxysteroid 17-beta dehydrogenase 12b |
| ***hsd20b2*** | Hydroxysteroid 20-beta dehydrogenase 2 |
| ***hsd3b1*** | Hydroxysteroid 3-beta dehydrogenase 1 |
| ***irf9y*** | Interferon regulatory factor 9, Y-linked |
| ***lhcgr*** | Luteinizing hormone/choriogonadotropin receptor |
| ***lrh (nr5a2)*** | Liver receptor homolog 1 |
| ***nanos2*** | Nanos C2HC-type zinc finger 2 |
| ***nanos3*** | Nanos C2HC-type zinc finger 3 |
| ***nr5a2*** | Nuclear receptor subfamily 5 group A member 2 |
| ***piwil1*** | Piwi-like RNA-mediated gene silencing 1 |
| ***piwil2*** | Piwi-like RNA-mediated gene silencing 2 |
| ***pgr*** | Progesterone receptor |
| ***rec8*** | Meiotic recombination protein REC8 |
| ***rspo1*** | R-spondin 1 |
| ***sdy*** | Sexually dimorphic on the Y chromosome |
| ***sf1 (nr5a1)*** | Steroidogenic factor 1 |
| ***sox3*** | SRY-box transcription factor 3 |
| ***sox3y*** | SRY-box transcription factor 3, Y-linked |
| ***sox9*** | SRY-box transcription factor 9 |
| ***star2*** | Steroidogenic acute regulatory protein 2 |
| ***sycp3*** | Synaptonemal complex protein 3 |
| ***tsp1*** | Thrombospondin 1 |
| ***wt1*** | Wilms tumor 1 |
| ***wnt4*** | Wingless-type MMTV integration site family member 4 |
| ***zp3f.2*** | Zona pellucida glycoprotein 3 family member 2 |
| ***zpd*** | Zona pellucida glycoprotein D |
