# Supplementary material for: Comparative RNA-Seq Analysis of Differentially Expressed Genes in the Testis and Ovary of Mudskipper, Boleophthalmus pectinirostris
Source: Animals (Basel). 2026 Jan 5;16(1):150. doi: 10.3390/ani16010150 (PMC12784797; doi:10.3390/ani16010150)
Supplement: Supplementary file 1 [file animals-16-00150-s001.zip › Table S1-S4.docx]

**Table S1. Summary of RNA-seq quality metrics.**

| Sample | RawData(bp) | CleanData(bp) | Q20(%) | Q30(%) | GC(%) | Total reads | Total mapped reads(%) | Mapped to exon of genome |
| --- | --- | --- | --- | --- | --- | --- | --- | --- |
| O1 | 6522003600 | 6447400633 | 97.91% | 93.92% | 48.69% | 43240850 | 40688258 (94.10%) | 38450597 (94.50%) |
| O2 | 6320952000 | 6180459375 | 97.99% | 94.01% | 48.20% | 41885962 | 39397278 (94.06%) | 37089291 (94.14%) |
| O3 | 5691612600 | 5527597685 | 97.66% | 93.32% | 47.49% | 37646352 | 35064470 (93.14%) | 32923748 (93.89%) |
| T1 | 6505361700 | 6355531271 | 97.90% | 93.99% | 46.39% | 43042714 | 39454291 (91.66%) | 34772466 (88.13%) |
| T2 | 6345744000 | 6194743074 | 97.72% | 93.50% | 46.47% | 41925224 | 38233928 (91.20%) | 33938877 (88.77%) |
| T3 | 6083598900 | 5960817633 | 97.87% | 93.77% | 47.02% | 40226622 | 36913141 (91.76%) | 32967745 (89.31%) |

**Table S2. Summary of gene expression statistics.**

| Sample | Refer genes | Sequenced refer genes(%) | Novel genes | Sequenced novel genes(%) | Total genes | Sequenced total genes(%) |
| --- | --- | --- | --- | --- | --- | --- |
| O1 | 22865 | 18260 (79.86%) | 4277 | 2030 (47.46%) | 27142 | 20290 (74.75%) |
| O2 | 22865 | 18624 (81.45%) | 4277 | 2199 (51.41%) | 27142 | 20823 (76.72%) |
| O3 | 22865 | 18634 (81.50%) | 4277 | 2198 (51.39%) | 27142 | 20832 (76.75%) |
| T1 | 22865 | 21820 (95.43%) | 4277 | 3565 (83.35%) | 27142 | 25385 (93.53%) |
| T2 | 22865 | 21708 (94.94%) | 4277 | 3549 (82.98%) | 27142 | 25257 (93.06%) |
| T3 | 22865 | 21718 (94.98%) | 4277 | 3527 (82.46%) | 27142 | 25245 (93.01%) |

**Table S3. Primers used for the gene expression analysis.**

| Gene name | Primer name | Primer sequence(5′→3′) | Length (bp) | GenBank No. |
| --- | --- | --- | --- | --- |
| *dmrt1* | Forward | TAGCCGAGAGACAGAGGGTC | 228 | XM_020919196.1 |
| *dmrt1* | Reverse | AAGATGACATGGGCCGACTC |  |  |
| *gdf9* | Forward | ATGACAGCGTCCCGTTTCTT | 229 | XM_020928753.2 |
| *gdf9* | Reverse | TGTGTTCGGGCTTGATCCTC |  |  |
| *foxl2* | Forward | CCTCACGCTGTCCGGTATTT | 220 | XM_020917494.2 |
| *foxl2* | Reverse | GCGCCTTCTGCGATAGTTTC |  |  |
| *amhr2* | Forward | TGGGCCAAACAGCTGTCATT | 245 | XM_055160180.1 |
| *amhr2* | Reverse | CACTGGTCCCCAACTCGTTT |  |  |
| *ctnnb1* | Forward | AGACCTGGGCTTGGACATTG | 238 | XM_020937095.2 |
| *ctnnb1* | Reverse | TTGGTTGGAATCACCGGGAG |  |  |
| *cyp17a1* | Forward | GTCAACCATCACACACACGC | 223 | XM_020921194.2 |
| *cyp17a1* | Reverse | CCTCTGCGCAAATGATGCTC |  |  |
| *hsd3b1* | Forward | CCAAACCCTAGTGGTGACCC | 224 | XM_020920393.2 |
| *hsd3b1* | Reverse | CCATCTGCCATATGCCCCAA |  |  |
| *hsd11b1* | Forward | AATGGAGTGCGCCAAGTTTTC | 221 | XM_020940124.2 |
| *hsd11b1* | Reverse | GGCCATGTCTCCTGCAATGTA |  |  |
| *piwi2* | Forward | ACCCCCAGGTCCATTTTGAG | 210 | XM_055161833.1 |
| *piwi2* | Reverse | GCACAGTGTTGATGCGCTTT |  |  |
| *gsdf* | Forward | TTTGAGTCTACAGGCCGAGC | 235 | XM_020925283.2 |
| *gsdf* | Reverse | AAATGTGAGACTCGCAGGGT |  |  |
| *figla* | Forward | GGCTGCGACCGAGTACATAA | 233 | XM_020926898.2 |
| *figla* | Reverse | CCTGCAAGACCAGTCTCGTT |  |  |
| *bmp15* | Forward | AACCCCCAAAAACCGCTGTA | 241 | XM_020917811.2 |
| *bmp15* | Reverse | GAGGGGGAGGTATTTGAGCG |  |  |
| *actin* | Forward | GGCATCAGGGAGTGATGGTT | 230 | XM_020927288.2 |
| *actin* | Reverse | GTTGGCTTTGGGGTTCAGTG |  |  |
| *β-2-microglobulin* | Forward | GATCCGGGACTTTTCGGCAA | 243 | XM_020938238.2 |
| *β-2-microglobulin* | Reverse | CCAGACGTAGTCCGTGACC |  |  |
| *ef1a* | Forward | GGCCAGATCAATGCGGGAT | 218 | XM_020932525.2 |
| *ef1a* | Reverse | CGACCAAGGGGAGGGAAATC |  |  |
| *Amh-splice* | Forward | CACCATGATGGCAATACTGA | 331 | XM_020940051.2 |
| *Amh-splice* | Reverse | GTTGTATAGGACCAAAGGTC |  |  |

**Table S4.** **Functional annotation of differentially expressed sex-related genes.**

| Symbol | Accession | log2(fc) | PValue | FDR | Description |
| --- | --- | --- | --- | --- | --- |
| *cyp19a1b* | XM_055155090.1 | 12.650 | 0.001902 | 0.003184 | brain aromatase |
| *lhcgra* | XM_020921083.2 | 9.920 | 1.57E-07 | 4.05E-07 | luteinizing hormone/choriogonadotropin receptor |
| *sox1a* | XM_020936277.1 | 8.810 | 0.008143 | 0.01252 | SRY-box transcription factor 1a |
| *wnt4b* | XM_020928034.2 | 8.266 | 2.23E-13 | 9.36E-13 | wingless-type MMTV integration site family, member 4b |
| *hsd17b3* | XM_055161737.1 | 8.184 | 2.62E-06 | 5.98E-06 | hydroxysteroid (17-beta) dehydrogenase 3 |
| *hsd20b2* | XM_020922828.2 | 8.148 | 2.09E-10 | 6.99E-10 | hydroxysteroid (20-beta) dehydrogenase 2 |
| *hsd11b2* | XM_020934502.2 | 7.613 | 1.63E-10 | 5.49E-10 | hydroxysteroid (11-beta) dehydrogenase 2 |
| *gdf6a* | XM_020937576.2 | 7.571 | 5.53E-08 | 1.49E-07 | growth differentiation factor 6a |
| *gsdf* | XM_020925283.2 | 7.450 | 1.76E-125 | 4.21E-122 | gonadal somatic cell derived factor |
| *nanos2* | XM_055150795.1 | 7.334 | 8.66E-08 | 2.29E-07 | nanos homolog 2 |
| *cyp11b1* | XM_020928097.2 | 7.159 | 4.77E-34 | 7.30E-33 | cytochrome P450 11B, mitochondrial |
| *rspo1* | XM_020940482.2 | 7.116 | 6.21E-07 | 1.51E-06 | R-spondin 1 |
| *dmrt1* | XM_020919196.1 | 7.063 | 2.44E-49 | 8.85E-48 | doublesex and mab-3 related transcription factor 1 |
| *sox1b* | XM_055166071.1 | 6.927 | 1.61E-06 | 3.75E-06 | SRY-box transcription factor 1b |
| *cyp21a2* | XM_055163982.1 | 6.806 | 8.46E-70 | 9.69E-68 | steroid 21-hydroxylase |
| *foxl1* | XM_020935284.2 | 6.791 | 2.22E-08 | 6.20E-08 | forkhead box L1 |
| *star2* | XM_020936371.1 | 6.757 | 3.44E-12 | 1.32E-11 | steroidogenic acute regulatory protein 2 |
| *cyp26b1* | XM_020940263.1 | 6.615 | 1.90E-06 | 4.41E-06 | cytochrome P450 26B1 |
| *aldh1a2* | XM_020941332.2 | 6.599 | 3.58E-74 | 5.19E-72 | aldehyde dehydrogenase 1 family, member A2 |
| *sox9b* | MSTRG.6999.1 | 6.458 | 2.28E-21 | 1.63E-20 | transcription factor Sox 9b |
| *sox9a* | XM_020935559.2 | 6.121 | 2.83E-29 | 3.32E-28 | transcription factor Sox 9a |
| *pgr* | XM_055166855.1,  XM_055166854.1 | 6.050 | 1.05E-85 | 2.78E-83 | progesterone receptor |
| *lhcgrb* | XM_020921003.2 | 5.921 | 1.87E-22 | 1.44E-21 | lutropin-choriogonadotropic hormone receptor-like |
| *tsp1* | XM_020919304.2 | 5.900 | 1.64E-97 | 7.71E-95 | thrombospondin-1-like |
| *arα* | XM_055159496.1 | 5.592 | 2.24E-36 | 3.95E-35 | androgen receptor-like |
| *fshr* | XM_020940702.2 | 5.583 | 8.34E-44 | 2.20E-42 | follicle stimulating hormone receptor |
| *hsd11b2* | XM_020940147.2 | 5.454 | 8.78E-05 | 0.000171 | hydroxysteroid 11-beta-dehydrogenase 1-like protein |
| *hsd3b1* | XM_020920393.2 | 5.356 | 1.81E-32 | 2.53E-31 | hydroxy-delta-5-steroid dehydrogenase, 3 beta- and steroid delta-isomerase 1 |
| *esr2a* | XM_020941688.2,  XM_055149630.1 | 4.741 | 1.06E-63 | 8.75E-62 | estrogen receptor 2a, transcript variant X2 |
| *gdf6b* | XM_020934373.2 | 4.683 | 0.001836 | 0.003078 | growth differentiation factor 6b |
| *cyp17a1* | XM_020921194.2 | 4.619 | 2.15E-56 | 1.17E-54 | steroid 17-alpha-hydroxylase/17,20 lyase |
| *elof1* | XM_020938734.2 | 4.581 | 1.83E-21 | 1.31E-20 | elongation factor 1 |
| *cyp17a2* | XM_055156179.1 | 4.287 | 1.35E-14 | 6.20E-14 | steroid 17-alpha-hydroxylase/17,20 lyase |
| *sf1* | XM_055161610.1 | 4.223 | 0.005019 | 0.007958 | nuclear receptor subfamily 5 group A member 1 |
| *dmrt4* | XM_020938144.2 | 4.222 | 0.01096 | 0.01651 | doublesex and mab-3 related transcription factor 4 |
| *hsd11b1* | XM_020940124.2 | 4.194 | 2.21E-22 | 1.69E-21 | hydroxysteroid 11-beta-dehydrogenase 1-like protein |
| *dmrt4* | XM_055156703.1,  XM_055156704.1 | 4.170 | 0.000479 | 0.000859 | doublesex- and mab-3-related transcription factor A4 |
| *arβ* | XM_020933588.2 | 3.923 | 3.80E-28 | 4.17E-27 | androgen receptor-like |
| *dmrt2b* | XM_020924846.2 | 3.623 | 6.10E-06 | 1.34E-05 | doublesex and mab-3 related transcription factor 2b |
| *piwil1* | XM_020935456.2 | 3.428 | 6.66E-45 | 1.88E-43 | piwi-like RNA-mediated gene silencing 1 |
| *hsd17b12b* | XM_020927456.2 | 3.214 | 5.50E-35 | 8.94E-34 | hydroxysteroid (17-beta) dehydrogenase 12b |
| *esr1* | XM_020941303.2,  XM_055149311.1 | 3.033 | 3.72E-22 | 2.80E-21 | estrogen receptor 1 |
| *bmpr2* | XM_055160180.1 | 3.030 | 2.51E-24 | 2.16E-23 | bone morphogenetic protein receptor type-2 |
| *piwil2* | XM_055161833.1,  XM_055161834.1 | 2.769 | 5.68E-20 | 3.70E-19 | piwi-like RNA-mediated gene silencing 2 |
| *foxl2l* | XM_055165916.1 | 2.685 | 4.43E-11 | 1.57E-10 | forkhead box L2-like |
| *nr5a2a* | XM_020924867.2 | 2.375 | 0.002702 | 0.004433 | nuclear receptor subfamily 5, group A, member 2 |
| *βcatenin* | XM_020922342.2 | 2.306 | 1.47E-15 | 7.20E-15 | catenin, beta like 1 |
| *vasa* | XM_055164615.1,  XM_055164604.1,  XM_055164593.1 | 1.905 | 6.46E-13 | 2.62E-12 | DEAD (Asp-Glu-Ala-Asp) box polypeptide 4 |
| *wnt4a* | MSTRG.25663.1 | 1.515 | 0.132403 | 0.167801 | wingless-type MMTV integration site family, member 4a |
| *nanos3* | XM_020930385.2 | -4.916 | 5.87E-74 | 8.40E-72 | nanos homolog 3 |
| *zp3f.2* | XM_055161085.1 | -4.834 | 4.01E-46 | 1.21E-44 | zona pellucida glycoprotein 3f, tandem duplicate 2 |
| *hsd17b12a* | XM_055155788.1 | -4.288 | 3.75E-34 | 5.77E-33 | hydroxysteroid (17-beta) dehydrogenase 12a |
| *foxlhl* | XM_055157071.1 | -4.033 | 1.82E-44 | 4.94E-43 | forkhead box protein H1-like |
| *zpd* | XM_020935052.2 | -3.912 | 9.69E-71 | 1.18E-68 | zona pellucida glycoprotein d |
| *bmp15* | XM_020917811.2 | -3.901 | 1.66E-24 | 1.45E-23 | bone morphogenetic protein 15 |
| *sox3* | XM_020923387.2 | -3.884 | 3.07E-51 | 1.25E-49 | SRY-box transcription factor 3 |
| *gdf9* | XM_020928753.2 | -3.688 | 1.38E-32 | 1.95E-31 | growth differentiation factor 9 |
| *gdf3* | XM_055151919.1 | -3.221 | 1.04E-25 | 9.76E-25 | growth differentiation factor 3 |
| *cyp26a1* | XM_020918369.2 | -3.184 | 1.72E-22 | 1.33E-21 | cytochrome P450 26A1 |
| *hsd17b1* | XM_020924045.2 | -3.005 | 0.000149 | 0.000284 | hydroxysteroid (17-beta) dehydrogenase 1 |
| *foxl3* | XM_020924426.2 | -2.777 | 1.04E-14 | 4.82E-14 | forkhead box L3 |
| *hsd17b2* | XM_020927536.2,  XM_020927537.2 | -2.590 | 1.29E-10 | 4.40E-10 | hydroxysteroid (17-beta) dehydrogenase 2, transcript variant X1 |
