# Supplementary figures and images for: Comparative RNA-Seq Analysis of Differentially Expressed Genes in the Testis and Ovary of Mudskipper, Boleophthalmus pectinirostris
Source: Animals (Basel). 2026 Jan 5;16(1):150. doi: 10.3390/ani16010150 (PMC12784797; doi:10.3390/ani16010150)

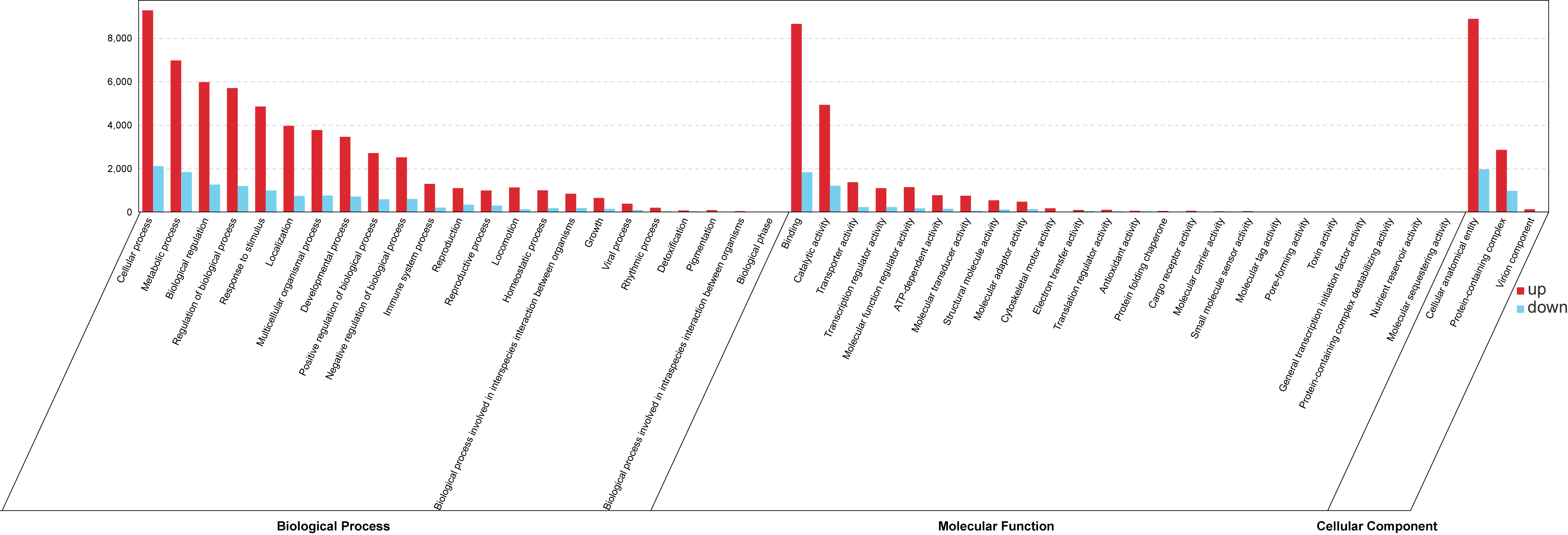

Supplement: Supplementary file 1 [file animals-16-00150-s001.zip › Figure S1 allGO classification .png]

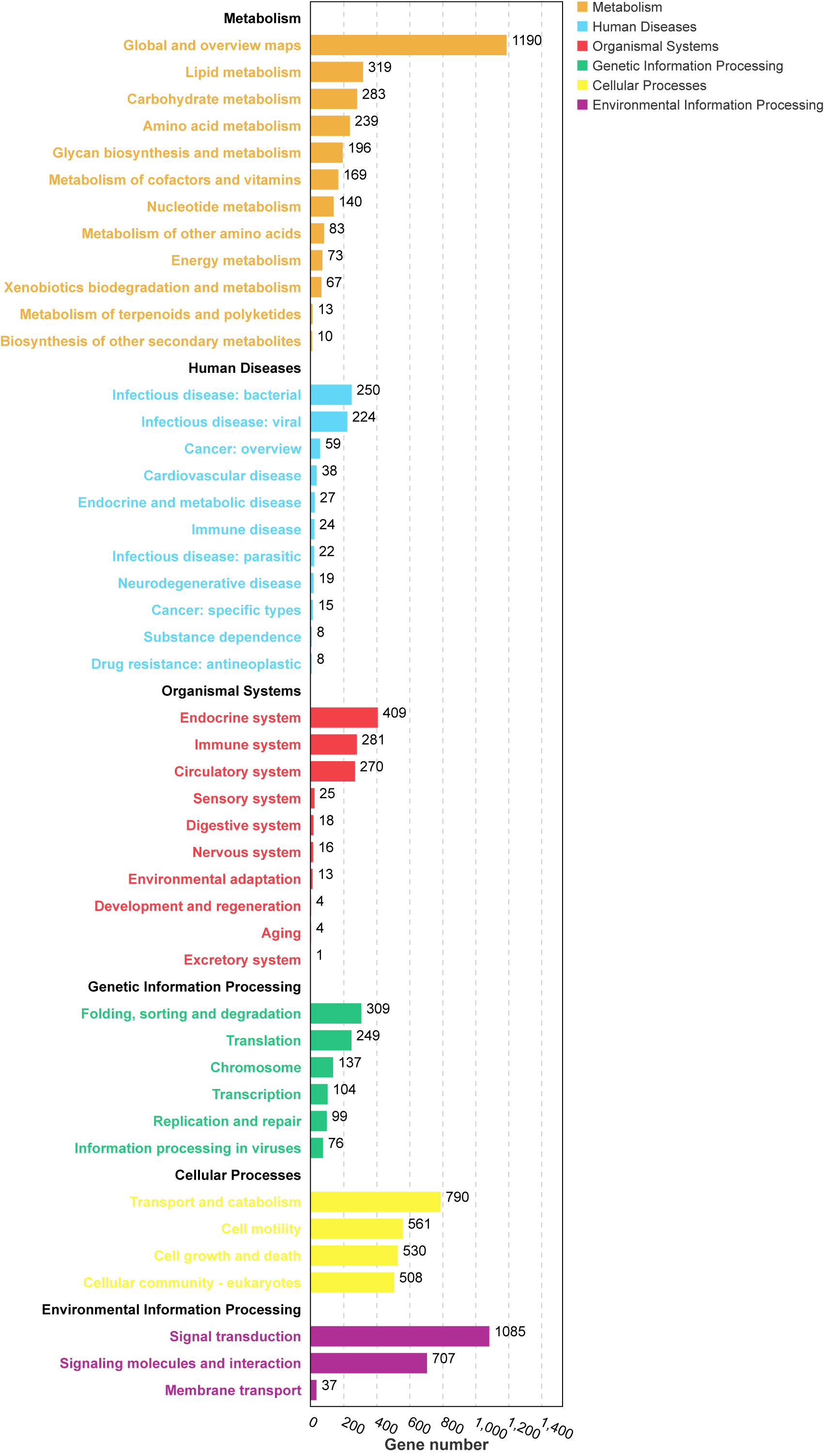

Supplement: Supplementary file 1 [file animals-16-00150-s001.zip › Figure S2 allKEGG classification .png]

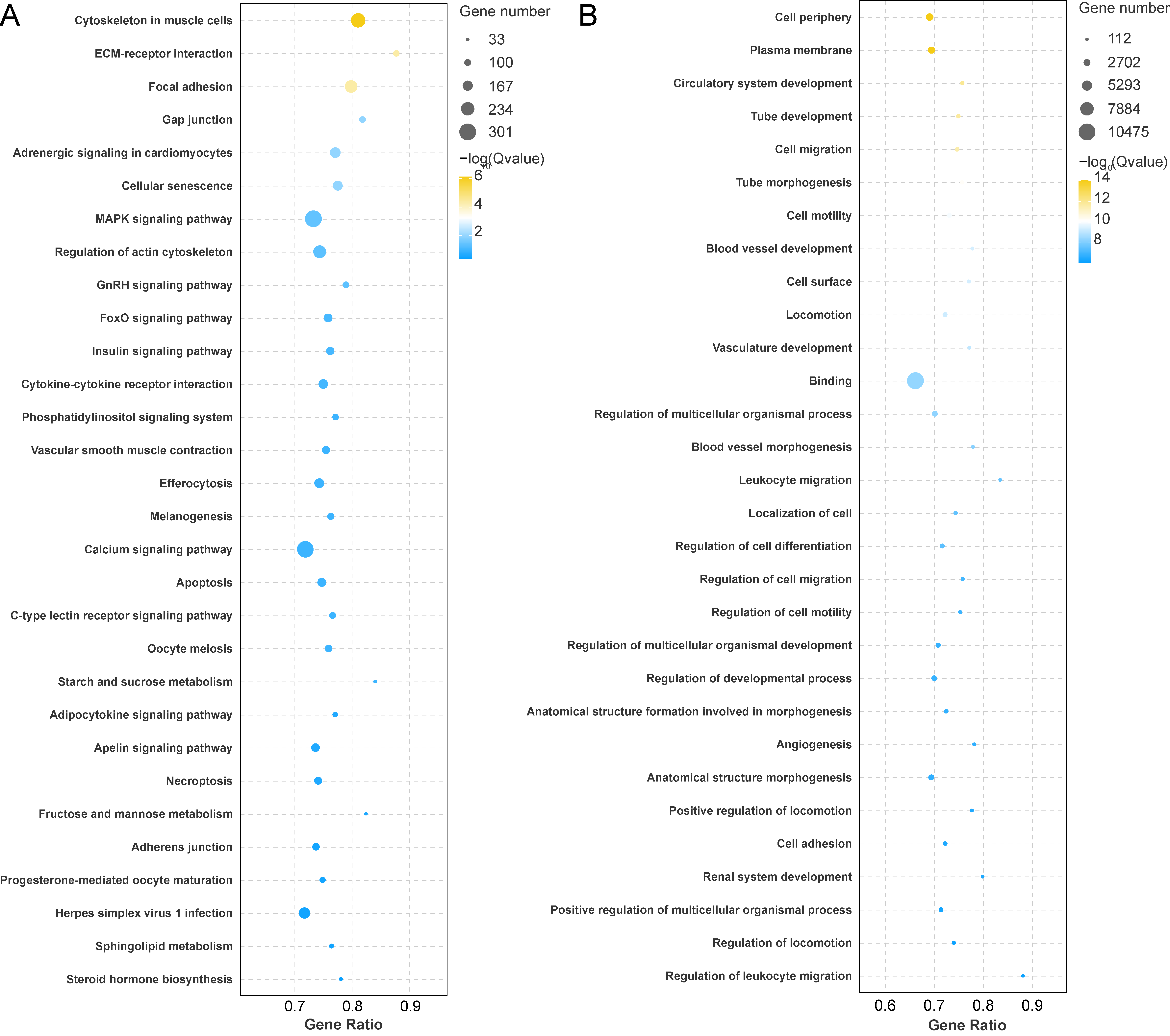

Supplement: Supplementary file 1 [file animals-16-00150-s001.zip › Figure S3 all diff GO KEGG changed.png]

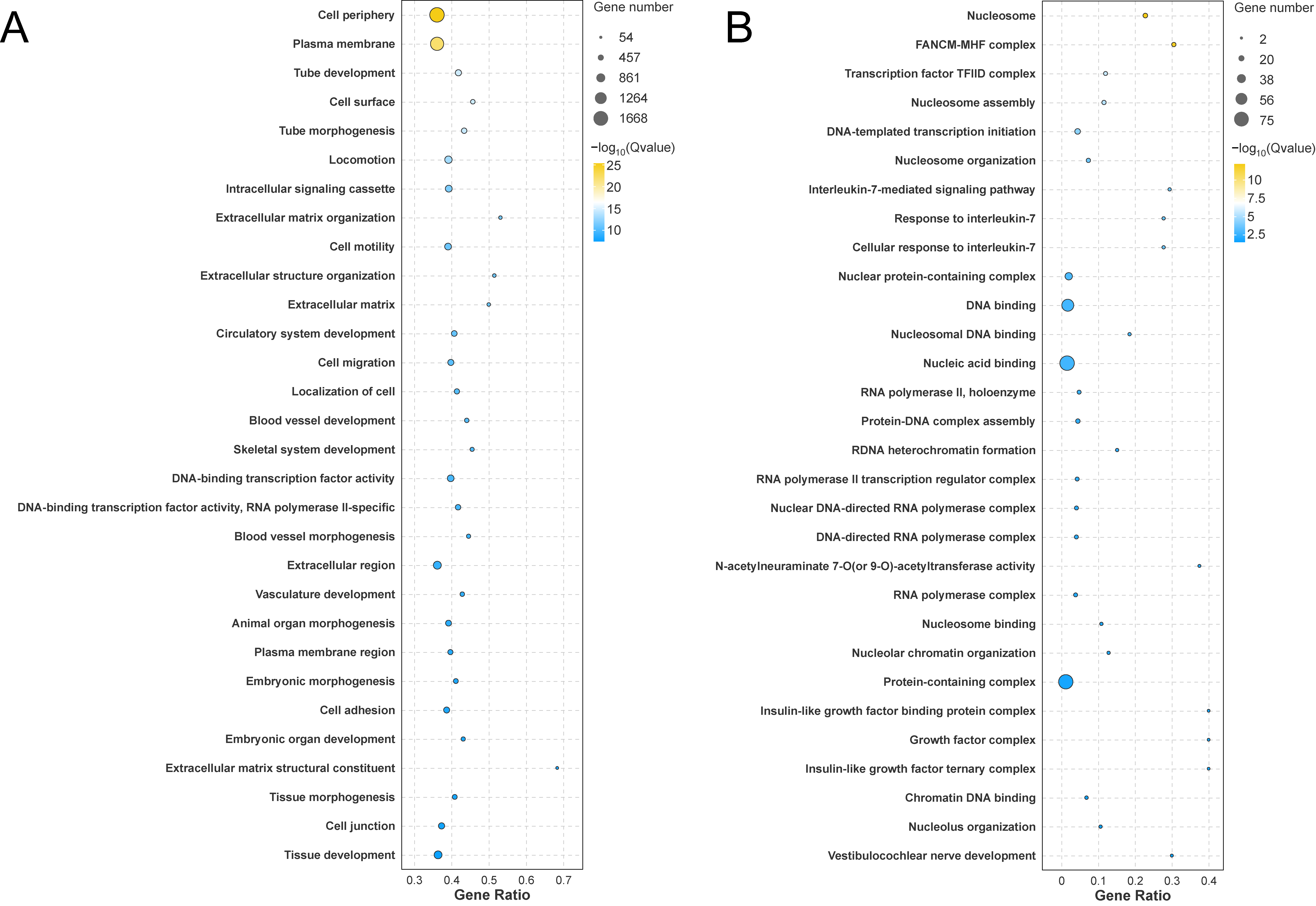

Supplement: Supplementary file 1 [file animals-16-00150-s001.zip › Figure S4 malefemale-specific-GO1 changed.png]

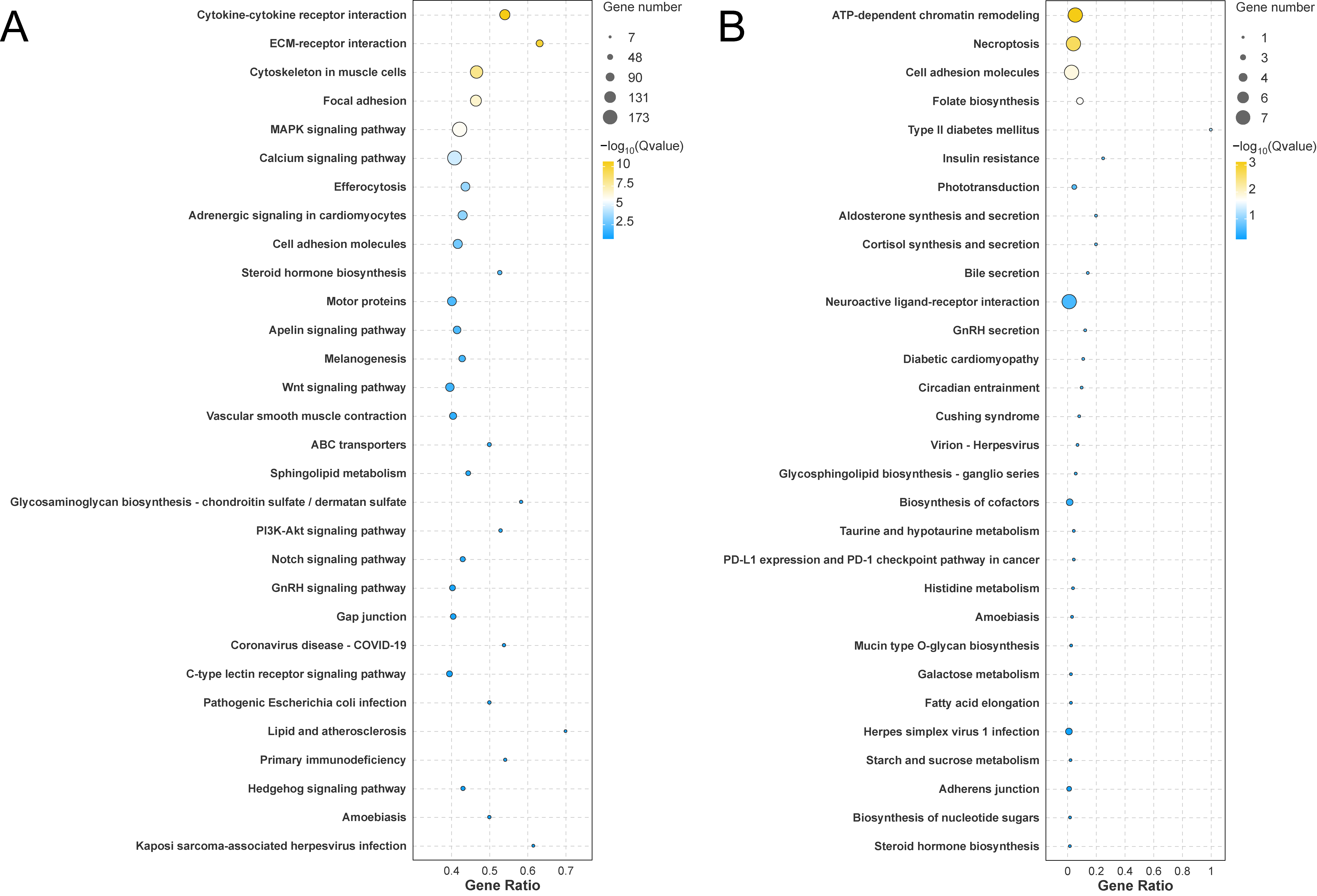

Supplement: Supplementary file 1 [file animals-16-00150-s001.zip › Figure S5 malefemale-specific-KEGG1 changed.png]

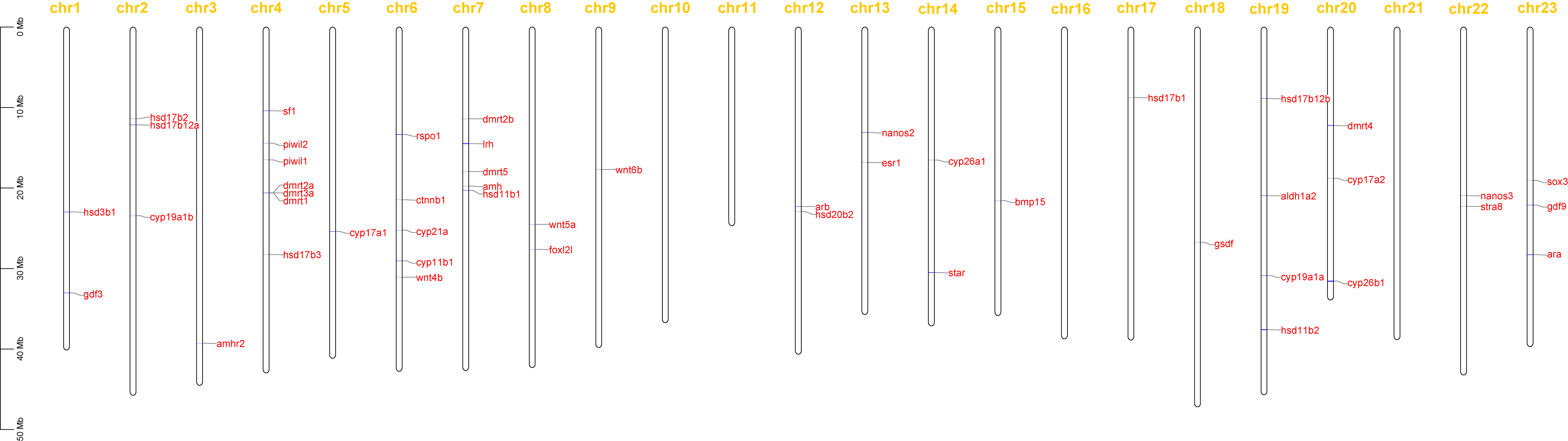

Supplement: Supplementary file 1 [file animals-16-00150-s001.zip › Figure S6 chr.png]

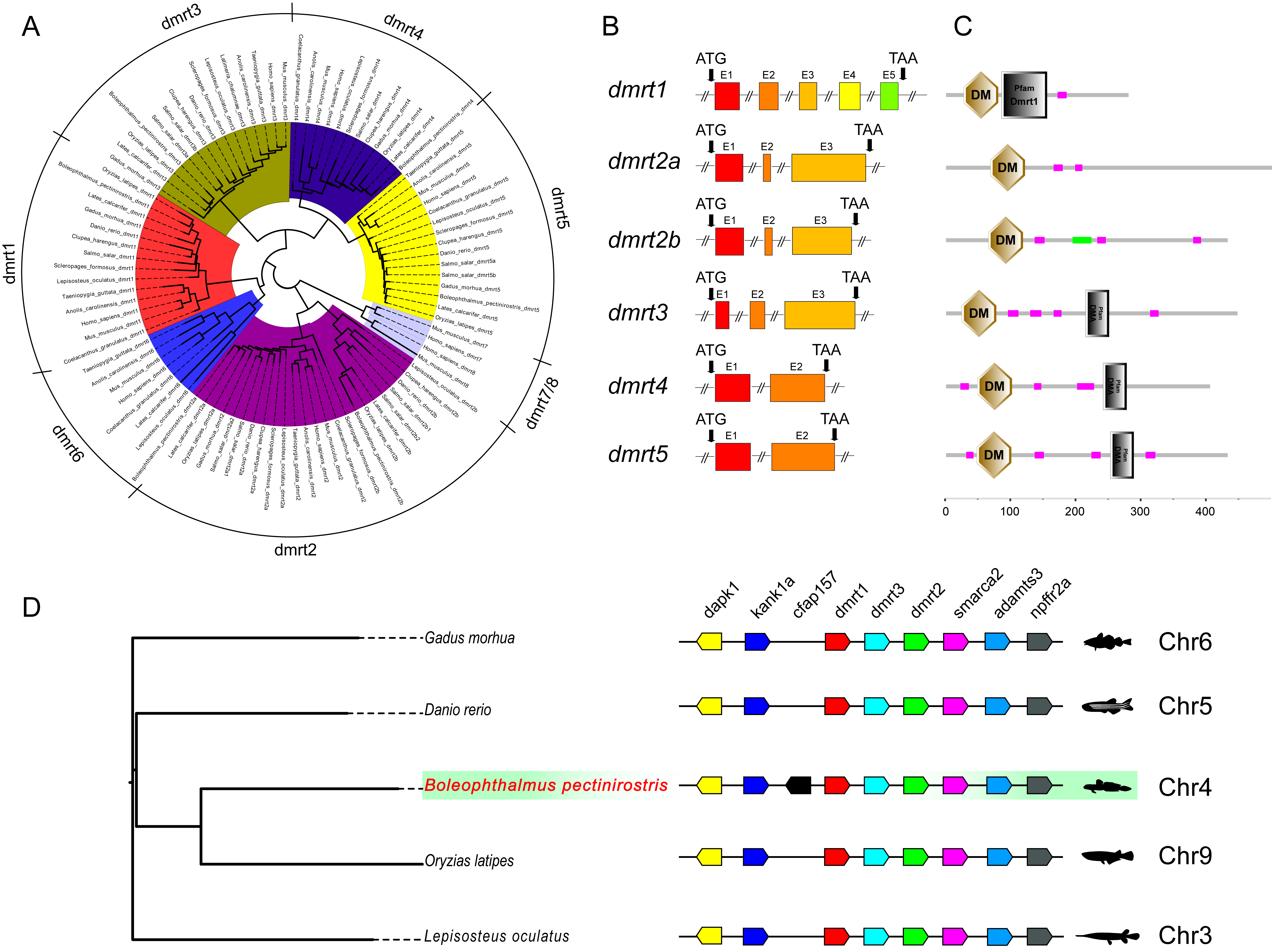

Supplement: Supplementary file 1 [file animals-16-00150-s001.zip › Figure S7 dmrt.png]

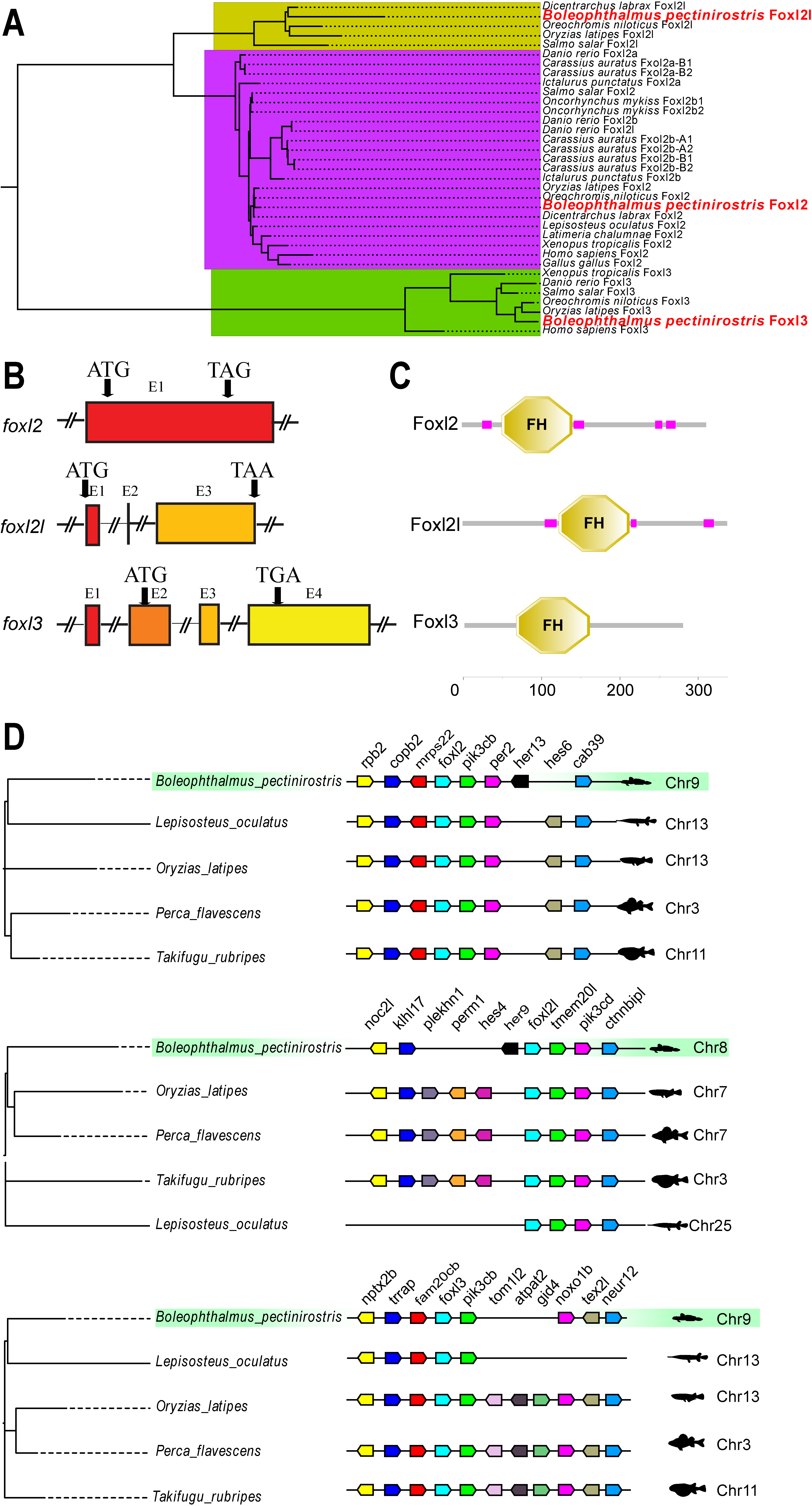

Supplement: Supplementary file 1 [file animals-16-00150-s001.zip › Figure S8 foxl changed.png]
